# KSRP improves pancreatic beta cell function and survival

**DOI:** 10.1038/s41598-024-55505-8

**Published:** 2024-03-13

**Authors:** Leticia Barssotti, Gabriela Moreira Soares, Emílio Marconato-Júnior, Bruna Lourençoni Alves, Kênia Moreno Oliveira, Everardo Magalhães Carneiro, Antonio Carlos Boschero, Helena Cristina Lima Barbosa

**Affiliations:** Obesity and Comorbidities Research Center (OCRC), Department of Structural and Functional Biology, Institute of Biology, University of Campinas (UNICAMP), Campinas, SP 13083864 Brazil

**Keywords:** KSRP, Pancreatic beta cell, Insulin secretion, Cell death, ER stress, Type 2 diabetes, Apoptosis, Stress signalling

## Abstract

Impaired insulin production and/or secretion by pancreatic beta cells can lead to high blood glucose levels and type 2 diabetes (T2D). Therefore, investigating new proteins involved in beta cell response to stress conditions could be useful in finding new targets for therapeutic approaches. KH-type splicing regulatory protein (KSRP) is a protein usually involved in gene expression due to its role in post-transcriptional regulation. Although there are studies describing the important role of KSRP in tissues closely related to glucose homeostasis, its effect on pancreatic beta cells has not been explored so far. Pancreatic islets from diet-induced obese mice (C57BL/6JUnib) were used to determine KSRP expression and we also performed in vitro experiments exposing INS-1E cells (pancreatic beta cell line) to different stressors (palmitate or cyclopiazonic acid—CPA) to induce cellular dysfunction. Here we show that KSRP expression is reduced in all the beta cell dysfunction models tested. In addition, when manipulated to knock down KSRP, beta cells exhibited increased death and impaired insulin secretion, whereas KSRP overexpression prevented cell death and increased insulin secretion. Taken together, our findings suggest that KSRP could be an important target to protect beta cells from impaired functioning and death.

## Introduction

Insulin resistance, along with impaired insulin production and/or secretion by beta cells, can lead to high blood glucose levels and type 2 diabetes mellitus (T2D). Obesity is a risk factor for the development of T2D, as elevated free fatty acids results in a lipotoxic state that can damage beta cells, increasing endoplasmic reticulum (ER) stress and oxidative stress, and compromising islet structure and function, resulting in a reduction of insulin synthesis^1,2^.

ER stress is the cellular response activated when there is impairment in ER functioning and consequent accumulation of misfolded proteins. As beta cells need constant changes in the synthesis capacity of their ER to increase insulin production and ensure its stocks inside the cell, they are even more susceptible to ER stress^3,4^. However, the exact mechanism leading to beta cell dysfunction in these cases is still unknown. Therefore, investigating new proteins modulated by beta cell stressors and their potential protective effect on cell function and survival is important while searching for new targets and therapeutic approaches.

KH-type splicing regulatory protein (KSRP), also named Far upstream element-binding protein 2, FUSE-binding protein 2, KHSRP, FUBP2, FBP2, and p75, is a protein that can interact either with RNA or DNA. KSRP is usually involved in gene expression due to its role in post-transcriptional regulation, participating in mechanisms such as alternative splicing, degradation of messenger RNA (mRNA), and primary processing and maturation of microRNAs^5–7^.

Some known KSRP targets are involved in cytoskeleton reorganization and insulin-stimulated glucose uptake^8,9^. KSRP knockout in mice leads to a decrease in triglycerides in the liver and induces white adipose tissue browning. Although there are studies describing the role of KSRP in tissues closely related to glycemic homeostasis, its effect on pancreatic beta cells has not been explored so far. Thus, we investigated KSRP expression in pancreatic islets from obese mice and in in vitro models of beta cell dysfunction. Moreover, we investigated the effect of in vitro knockdown and overexpression of KSRP on pancreatic beta cell function and survival.

Here we show that KSRP expression is decreased in obese mice islets and in in vitro dysfunctional beta cells. The knockdown of KSRP increased beta cell death and impaired insulin secretion, while overexpression prevented cell death and increased insulin secretion. These results are supported by modulations in INS2 gene expression and STX1A protein expression, involved in insulin production and granule extrusion, respectively. Therefore, our findings suggest that KSRP could be an important target to protect beta cells from impaired functioning and death.

## Material and methods

### Animals

C57BL/6JUnib male mice (28 days old) were purchased from the CEMIB/Unicamp and housed in the animal facility of the Department of Structural and Functional Biology/Unicamp (Campinas, Brazil). Animals had free access to a standard diet and water and were maintained in a temperature-controlled room (22 ± 2 °C) under a 12-h light–dark cycle. At 8 weeks old, mice were randomly assigned into two groups and received a standard chow diet (Ctl) or high-fat diet (HFD, 60% fat, Prag Soluções Biociências®, Jaú, SP, Brazil) for 16 weeks, with the body weight monitored weekly. The study was approved by the Institutional Ethics Committee on Use of Animals (CEUA-Unicamp/Protocol #5359-1/2019) and performed following relevant named guidelines and regulations. The study was carried out in compliance with the ARRIVE guidelines.

### Intraperitoneal glucose (ipGTT) and insulin (ipITT) tolerance tests

After sixteen weeks of HFD, mice were subjected to 12 h fasting to perform the ipGTT. The fasting blood glucose level was measured (time 0) with a glucometer. Mice received an intraperitoneal injection of glucose (2 g/kg), and glycemia was measured at 15, 30, 60, 90, and 120 min. Five days later, mice were subjected to 4 h fasting for the ipITT, and the glycemia was measured before (time 0) and 3, 6, 9, 12, 15, and 18 min after the intraperitoneal administration of 1 U/kg of insulin. The area under the curve (AUC) of blood glucose during the ipGTT and ipITT was calculated using GraphPad Prism 8 (San Diego, CA, USA).

### Pancreatic islet isolation

To assess gene expression, islets were isolated by collagenase digestion of the pancreas. Mice were anesthetized with isoflurane and euthanized by decapitation. The pancreas was inflated with 2.5 ml of Hanks balanced salt solution containing 0.8 mg collagenase/ml, excised, and incubated in a shaker at 37 °C for 10 min. Islets were then selected with a micropipette under a microscope to exclude exocrine pancreas-contaminating tissues^10,11^.

### INS-1E cell culture

INS-1E cells were cultured in RPMI-1640 medium (10-040CV, Corning), supplemented with 11.1 mmol/l glucose (G1008.01.AG, Synth), 5% fetal bovine serum (Vitrocell, Brazil), 10 mmol/l N-(2-Hydroxyethyl)piperazine-N′-(2-ethanesulfonic acid) (HEPES, H4034, Sigma), 100 U/ml penicillin/streptomycin (17-602F, Lonza), 1 mmol/l sodium pyruvate (P5280, Sigma), and 50 µmol/l 2-mercaptoethanol (M3148, Sigma), and kept under standard conditions (5% CO_2_ and 37 ºC).

### Palmitate and CPA treatments

INS-1E cells were cultured in a 24-well plate until 70% confluence. Cells were then incubated with RPMI-1640 medium containing 11.1 mmol/l glucose and supplemented with 10 mmol/l HEPES, 100 U/ml penicillin, 1 mmol/l sodium pyruvate, 50 µmol/l 2-mercaptoethanol, 1% bovine serum albumin (BSA, A7030, Sigma), and 0.5 mmol/l of palmitic acid (P5585, Sigma). To prepare the BSA-palmitate saturated fatty acid complex, the medium was heated in a water bath at 55 ºC for 25 min and then cooled to room temperature, filtered, and added to the cell. After 12 h, 16 h, and 24 h the cells were washed with PBS and collected for protein or mRNA quantifications. The control group was exposed to ethanol (vehicle). Cyclopiazonic acid (CPA, C1530, Sigma) was first diluted in dimethyl sulfoxide (DMSO, D8418, Sigma) solution and then added in the corresponding concentration (6.25 µmol/l, 12.5 µmol/l or 25 µmol/l) to the cell culture medium. After 12 h, 16 h, and 24 h the cells were washed with PBS and collected for protein quantifications. The control group was exposed only to DMSO (vehicle).

### siRNA transfection

INS-1E cells were seeded in 24-well culture plates until 70% confluence and incubated for 16 h in supplemented RPMI 1640 medium without antibiotic, Opti-MEM Reduced Serum Medium (Gibco®, 31985-070, Life Technologies), Lipofectamine™ RNAiMAX (13778-150, Invitrogen), and 70 nM ON-TARGETplus SMARTpool siRNA KSRP (Dharmacon) or AllStars Negative Control siRNA (1027281, Qiagen). The transfection medium was then replaced by the INS-1E regular culture medium and after 48 h samples were collected for RNA extraction or western blot analysis.

### Bacterial transformation and plasmid DNA extraction and purification

The pEGFP-C1 plasmid (control) was incubated with NEB Stable Competent *E. coli* bacteria subjected to heat shock for DNA internalization. 2xYT medium (peptone from meat, 91249, Sigma; yeast extract, Y1625, Sigma; and sodium chloride, C1060.01. AH, Synth) was used to promote bacteria recovery and growth. The plasmid pEGFP1-6XHis-FLKSRP (which we will refer to as pEGFP-C1-KSRP) was developed in DH5alpha bacteria by Douglas Black's Laboratory (Addgene #23001) and donated for this research. The bacteria were plated in agar (and selected by their resistance to antibiotic kanamycin), incubated overnight at 37 ºC, and single colonies were grown in a liquid 2xYT medium (overnight at 37 °C and 200 rpm). For the extraction and purification of the plasmid, the QIAprep® Spin Miniprep Kit (27106, Qiagen) was used according to the manufacturer’s protocol.

### Plasmid transfection

INS-1E cells grown in a 24-well plate after reaching 70% of confluence were incubated for 16 h in supplemented RPMI 1640 medium without antibiotic, Opti-MEM Reduced Serum Medium (Gibco®, 31985-070, Life Technologies), Lipofectamine™ 3000 Transfection Reagent (L3000-015, Invitrogen), and 500 ng of pEGFP-C1 plasmid (control) or pEGFP-C1-KSRP per well. The transfection was carried out according to the manufacturer's instructions. For cell death protocol the cells were grown in a 96-well plate and 100 ng of the plasmid was used.

### Western blot

Cells were washed with PBS 1 × and incubated at 100 °C for 5 min in Laemmli 5X buffer (0.1% bromophenol blue, sodium phosphate 1 M, glycerol 50%, SDS 10%). The proteins were separated by sodium dodecyl sulfate–polyacrylamide gel electrophoresis (SDS-PAGE), along with protein molecular weight standards (PageRuler, 26619, ThermoFisher). After electrophoresis, liquid transfer with 20% methanol to nitrocellulose membrane (BioRad, with 0.22 μm pore) was performed, and to block non-specific binding the membrane was incubated with TBS-T buffer (50 mM Tris pH 7.5, NaCl 150 mM, 0.1% Tween 20) containing 5% non-fat dry milk, during 90 min at room temperature. Membranes were incubated with specific primary antibodies: KSRP (HPA034739, Sigma), phospho-eukaryotic translation initiation factor 2α (p-eIF2α, ab32157, Abcam), C/EBP homologous protein (CHOP, ab11419, Abcam), syntaxin 1A (STX1A, sc-12736, Santa Cruz Biotechnology), immunoglobulin binding protein (GRP78/BiP, ab21685, Abcam), inositol-requiring enzyme type 1 (IRE1, ab37073, Abcam), and the housekeeping glyceraldehyde-3-phosphate dehydrogenase (GAPDH, G9545, Sigma). After washing with TBS-T buffer, membranes were incubated with anti-IgG polyclonal antibody conjugated with peroxidase (dilution 1:10,000 in TBS-T with 3% non-fat dry milk) for 90 min. Protein bands were visualized using the Amersham Imager 600 (GE Healthcare, Little Chalfont, UK) with chemiluminescence reagents. The intensity and quantification of the bands were evaluated by densitometry (ImageJ, Bethesda, USA). Normalization of protein content was performed using GAPDH and the results were presented as fold changes of their respective controls.

### Quantitative PCR

Isolated mice pancreatic islet and INS1-E cells were lysed in Trizol solution (15596018, Ambion by Life Technologies) and the total RNA was extracted according to the manufacturer's protocol. Spectramax i3 (Molecular Devices) was used to measure RNA concentration and complementary DNA (cDNA) was synthesized with 1 μg of RNA, using the High Capacity cDNA Reverse Transcription kit (4368813, Applied Biosystems). The quantitative PCR reaction was performed using Fast SYBR® Green PCR Master Mix (4385612, Applied Biosystems) in the 7500 Fast Real-Time PCR System (Applied Biosystems). Primer sequences used for real-time PCR assays were as follows:

KSRP (forward 5’ GCCTGTGAGATGGTGATGGAC 3’, reverse 5’ GAAGACTCTGAAGGAGGTCATTG 3’), insulin 2 (INS2, rat, forward 5’ CCAGCTAAGACCTCAGGGACT 3’, reverse 5’ CTGGAAGATAGGCTGGGTTGAG 3’), INS2 (mice, forward 5’ GTCAAGCAGCACCTTTGTGG 3’, reverse 5’ CAGTTGTGCCACTTGTGGGT 3’), pancreatic and duodenal homeobox 1 (PDX1, forward 5’ GAACCCGAGGAAAACAAGAGG 3’, reverse 5’ GTTCAACATCACTGCCAGCTC 3’), BiP (forward 5’ ACTTGGGGACCACCTATTCCT 3’, reverse 5’ ATCGCCAATCAGACGCTCC 3’), ATF4 (forward 5’ GTTGGTCAGTGCCTCAGACA 3’, reverse 5’ CATTCGAAACAGAGCATCGA 3’), XBP1 (forward 5’ GAGTCCGCAGCAGGTG 3’, reverse 5’ GCGTCAGAATCCATGGGA 3’), CHOP (forward 5’ CTGGAAGCCTGGTATGAGGAT 3’, reverse 5’ CAGGGTCAAGAGTAGTGAAGGT 3’) and hypoxanthine phosphoribosyltransferase (HPRT, forward 5’ GGTTAAGCAGTACAGCCCCA 3’, reverse 5’ TCCAACACTTCGAGAGGTCC 3’). The analyses were performed using the 2^-ΔΔCt^ method and the HPRT as the constitutive gene.

### Cell death

INS-1E cells cultured in a 96-well plate (90% confluent) were costained with the DNA intercalants Hoechst 33,342 (H3570, ThermoFisher) and propidium iodide (P1304MP, ThermoFisher) at a concentration of 0.05 μg/μl for 15 min at 37 ºC. The percentage of necrotic/apoptotic cells was determined using the High Content Imaging System (ImageXpress; Molecular Devices) through the Live and Dead module of MetaXpress Software (Molecular Devices). Nine fields per well (each well in triplicate) were captured, and masks were applied for the DAPI wavelengths (excitation at 350 nm and emission at 470 nm; for Hoechst) and Texas red (excitation at 596 nm and emission at 615 nm; for propidium iodide).

### Insulin secretion

INS1-E cells grown on 24-well plates and transfected with siRNA or plasmid (90% confluent) were incubated for 1 h at 37 °C with Krebs buffer (115 mM NaCl, 5 mM KCl, 2.56 mM CaCl_2_, 1 mM MgCl_2_, 10 mM NaHCO_3_, 15 mM HEPES) without glucose, supplemented with 0.3% fatty acid-free bovine serum albumin (BSA, A7030, Sigma), and balanced with a mixture of 95% O_2_/5% CO_2,_ pH 7.4. The solution was replaced by Krebs buffer containing different glucose concentrations (2.8 and 22.2 mM) for 1 h. Insulin supernatant was measured by radioimmunoassay^12^. The cells were collected using urea/thiourea buffer for total protein measurement. Insulin secretion was normalized by total protein.

### Statistical analysis

The data were submitted to the Shapiro–Wilk normality test and variables with parametric distribution were analyzed by student’s *t* test (only two groups) or One-Way ANOVA with Dunnett's post-hoc test (three or more groups). Non-parametric data were analyzed by the Mann–Whitney test (two groups) or Kruskal–Wallis with Dunn’s post-hoc test (three or more groups). CPA data (different concentrations and times) were submitted to the Two-way ANOVA test followed by Dunnett's post-test. The data are presented as the mean ± standard error of the mean (SEM) and were considered statistically significant if p ≤ 0.05. All analyses were performed using GraphPad Prism software version 8.0 for Windows.

## Results

### KSRP mRNA expression decreases in pancreatic islets from mice fed a high-fat diet for 16 weeks

**Figure 1:**
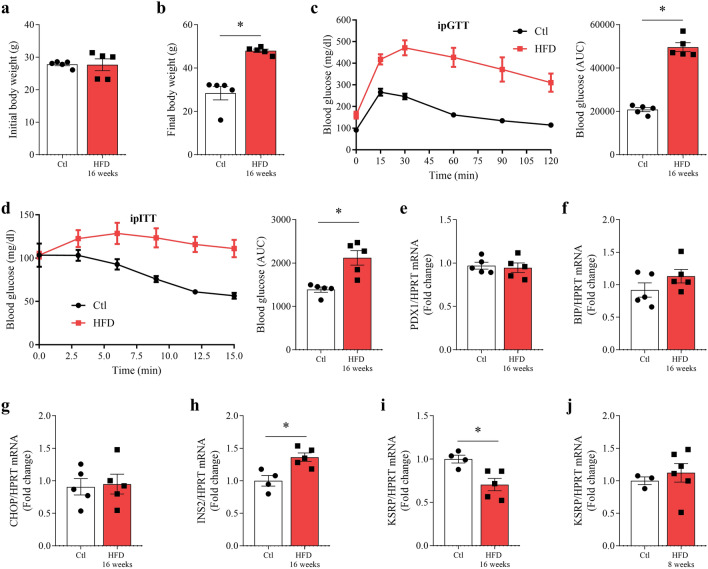
KSRP expression decreases in pancreatic islets from obese mice. Initial (**a**) and final (**b**) body weight. Blood glucose and area under the curve (AUC) during ipGTT (**c**) and ipITT (**d**). mRNA levels of PDX1 (**e**), BiP (**f**), CHOP (**g**), INS2 (**h**), and KSRP (**i**) normalized by HPRT in pancreatic islets from mice that were fed a standard chow diet (Ctl) or high-fat diet for 16 weeks (HFD). KSRP gene expression normalized by HPRT (**j**) was also measured after 8 weeks of HFD. The results were expressed as media ± SEM and were submitted to Student’s *t* test, except for body weight analysis (**a** and **b**), which were submitted to Mann–Whitney’s test (n = 4–5). *p ≤ 0.05. All experiments were performed in simplicate. *BiP,* binding immunoglobulin protein; *CHOP,* C/EBP homologous protein; *HPRT,* hypoxanthine phosphoribosyltransferase; *INS2,* insulin 2; *KSRP,* KH-type splicing regulatory protein; *PDX1,* pancreatic and duodenal homeobox 1.

Mice fed a high-fat diet over 16 weeks experienced an increase in body weight (p = 0.0079, Fig. 1b) compared to initial body weight at the start of the experiment (Fig. 1a). Obese mice also showed reduced glucose tolerance (p < 0.0001, Fig. 1c) as well as impaired insulin sensitivity (p = 0.0035, Fig. 1d), as determined by the increased AUC of blood glucose during ipGTT and ipITT (Fig. 1c and d). We did not observe differences in the gene expression of the PDX1 transcription factor (Fig. 1e), associated with beta cell identity and function, or in the ER-stress markers BiP and CHOP (Fig. 1f and g). However, mice fed a high-fat diet exhibited an increase in the insulin gene (INS2)-mRNA content (p = 0.0094, Fig. 1h), in agreement with the glucose homeostasis deregulations presented in the in vivo experiments. Additionally, we observed a decrease in KSRP mRNA content in islets from obese mice after 16 weeks diet (p = 0.0134, Fig. 1i) while after 8 weeks there were no difference in the KSRP expression (Fig. 1j).

### KSRP gene and protein expression are decreased in INS-1E cells exposed to palmitate for 24 h

**Figure 2:**
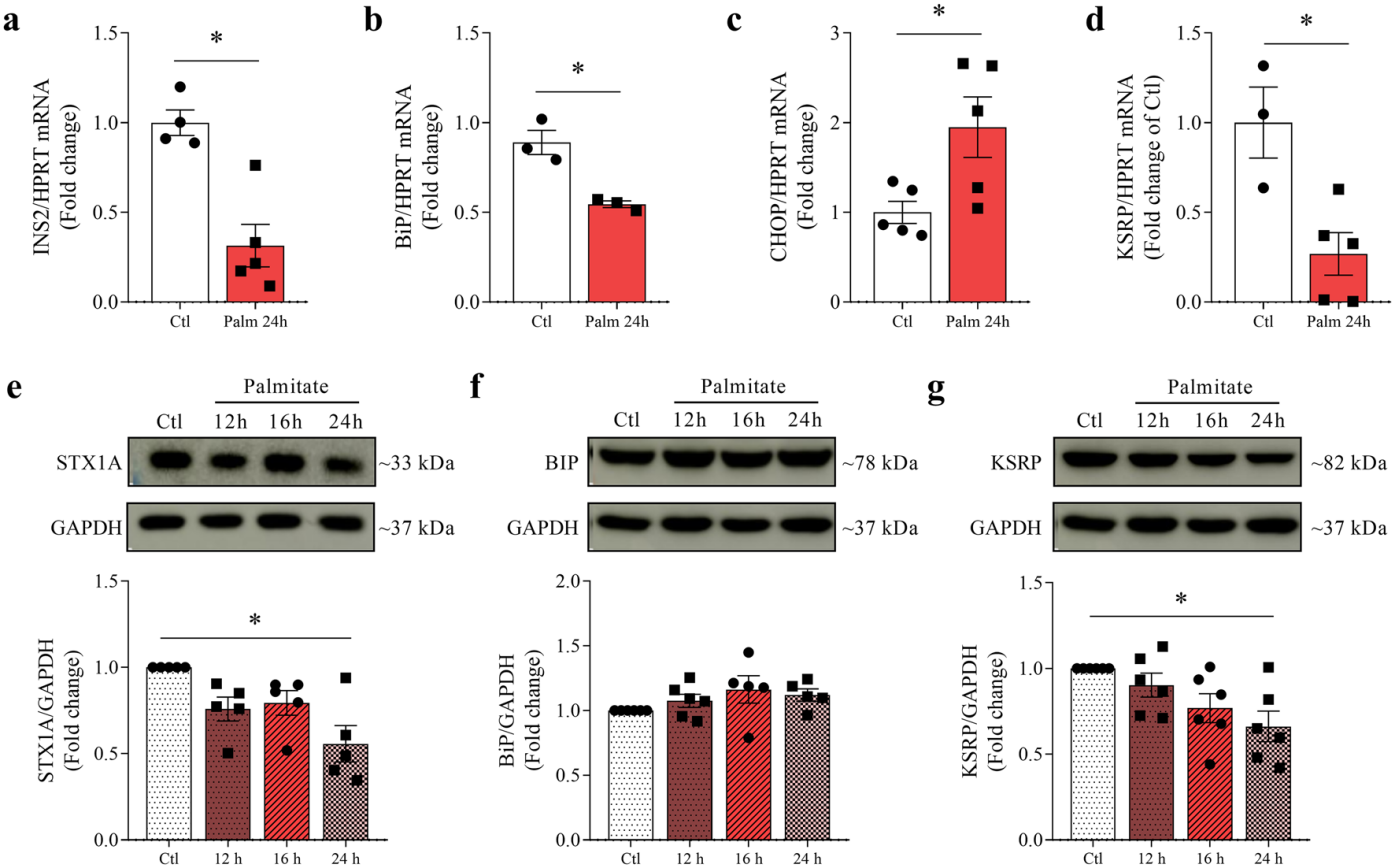
KSRP expression decreases in INS-1E cells exposed to palmitate. mRNA analysis of INS2 (**a**), BiP (**b**), CHOP (**c**), and KSRP (**d**) normalized by HPRT after 24 h treatment with 0.5 mmol/l of palmitate. The results were expressed as media ± SEM and were submitted to Student’s *t* test (n = 3–5). Western blot analysis of STX1A (**e**), BiP (**f**), and KSRP (**g**) proteins normalized by GAPDH after exposure to 0.5 mmol/l of palmitate (12 h, 16 h, or 24 h) in INS-1E cells. The results were expressed as media ± SEM and were submitted to Kruskal–Wallis followed by Dunn’s test (**e**), or One-way ANOVA (**f** and **g**) (n = 5–6). *p ≤ 0.05. All experiments were performed in simplicate. The membranes were cut prior to the exposure so that only the portion of gel containing the desired bands would be visualized. Original images for blots are presented in Supplementary Figs. S1–S4. *BiP,* binding immunoglobulin protein; *CHOP,* C/EBP homologous protein; *GAPDH,* glyceraldehyde-3-phosphate dehydrogenase; *INS2,* insulin 2; *HPRT,* hypoxanthine phosphoribosyltransferase; *KSRP,* KH-type splicing regulatory protein; *STX1A,* syntaxin 1A.

In vitro experimental models of pancreatic beta cell dysfunction associated with obesity and T2D progression involve exposing the insulin-producing beta cell to high concentrations of fatty acids. Therefore, we used palmitic acid, one of the most common saturated fatty acids in the diet and whose toxic effect on beta cells has been extensively studied^13–15^. mRNA analysis after 24 h exposure to palmitate also caused a decrease in the insulin gene expression (p = 0.0024, Fig. 2a), a decrease in the ER stress protein BiP (p = 0.0079, Fig. 2b), and an increase in the expression of death inducer CHOP (p = 0.0296, Fig. 2c). KSRP mRNA is decreased (p = 0.0141, Fig. 2d) after exposure to palmitate for 24 h. Protein expression of INS-1E cells exposed to palmitate for 12 h, 16 h, and 24 h shows that only after 24 h there is a decrease in STX1A (p = 0.0041, Fig. 2e) protein expression, which indicates impaired insulin granule extrusion caused by toxic effects of palmitate. BiP protein expression did not change (Fig. 2f), but KSRP decreased after 24 h (p = 0.008, Fig. 2g).

### KSRP protein expression is decreased in INS-1E cells with CPA-induced ER stress

**Figure 3:**
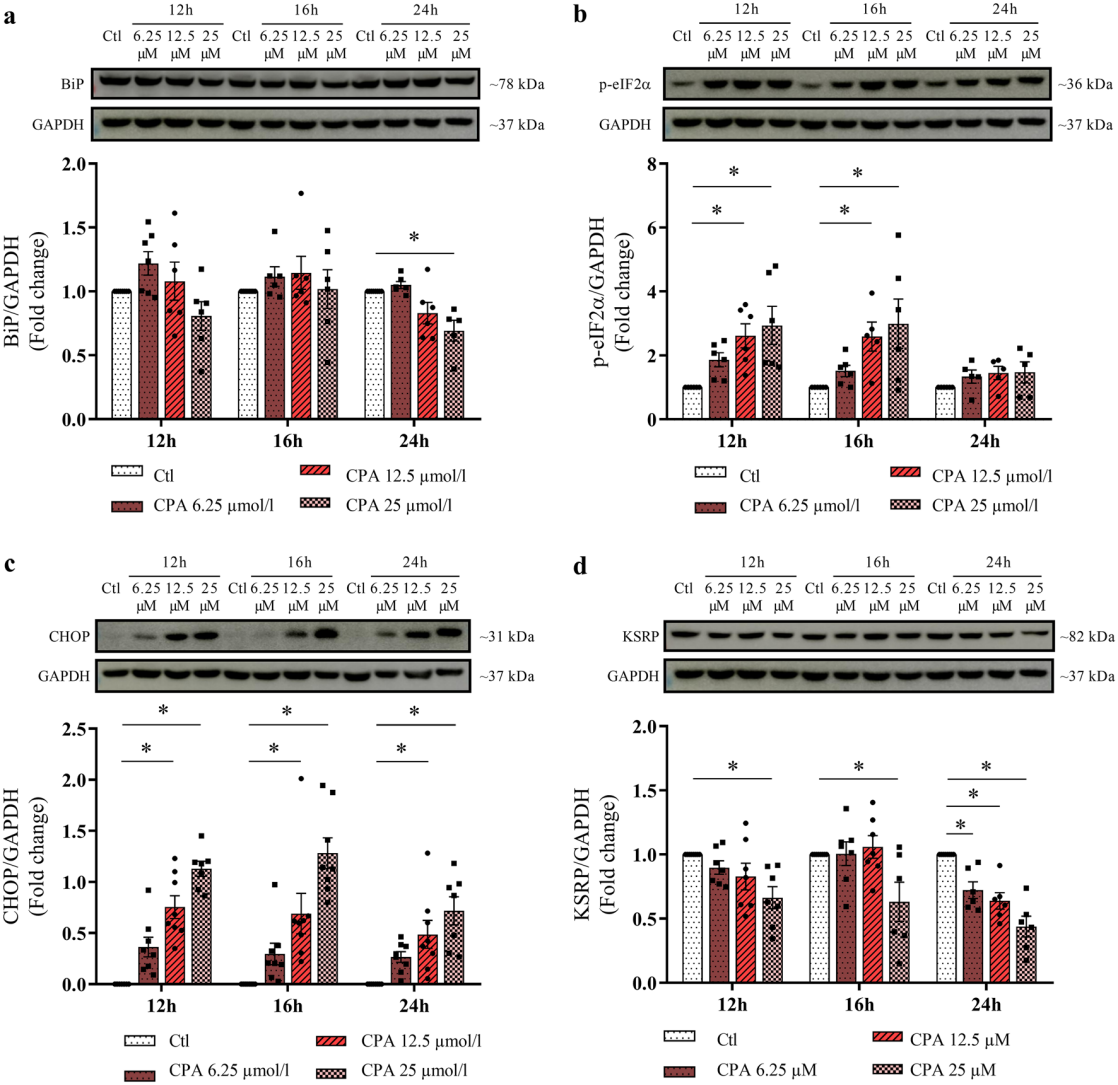
CPA-induced ER stress decreases KSRP expression in INS-1E cells. Western blot analysis of BiP (**a**), p-eIF2α (**b**), CHOP (**c**), and KSRP (**d**) normalized by GAPDH in INS-1E cells treated with 6.25 µmol/l, 12.5 µmol/l, or 25 µmol/l CPA for 12 h, 16 h, or 24 h. The results were expressed as media ± SEM and submitted to Two-way ANOVA test followed by Dunnet’s post-test (n = 5–8). *p ≤ 0.05. All experiments were performed in simplicate. The membranes were cut prior to exposure so that only the portion of gel containing the desired bands would be visualized. Original images for blots are presented in Supplementary Figs. S5–S13. *BiP,* binding immunoglobulin protein; *CHOP,* C/EBP homologous protein; *GAPDH,* glyceraldehyde-3-phosphate dehydrogenase; *KSRP,* KH-type splicing regulatory protein; *p-eIF2α,* eukaryotic translation initiation factor 2α*.*

CPA, a chemical inhibitor of the ER-calcium pump (SERCA), induces dysfunction in pancreatic beta cells more aggressively than palmitate. Calcium plays a key role in the functioning of the ER and the folding of newly synthesized proteins requires calcium as a coenzyme. Therefore, inhibition of calcium uptake by the ER induces stress, activating all components of the UPR, CHOP expression, and apoptosis^16,17^. Thus, we induced beta cell dysfunction through CPA-induced ER stress to evaluate its impacts on the KSRP expression. First, we analyzed the protein content of ER-stress markers. BiP was significantly decreased after 24 h exposure to 25 µmol/l of CPA (p = 0.0435, Fig. 3a). p-eIF2α increased after incubation with 12.5 µmol/l and 25 µmol/l of CPA for 12 h (p = 0.0067 and p = 0.0010, respectively; Fig. 3b) and for 16 h (p = 0.0115 and p = 0.0007, respectively; Fig. 3b), but did not change after 24 h. After 12 h, 16 h, and 24 h of exposure to 12.5 µmol/l and 25 µmol/l of CPA, CHOP protein expression was significantly increased (p = 0.0055 for Ctl vs. CPA 12,5 µmol/l; and p < 0,0001 for all the other comparisons, Fig. 3c), confirming ER-stress induction in all exposure times and concentrations. KSRP protein expression decreased after 12 h and 16 h incubation with 25.0 µmol/l of CPA (p = 0.0039 and p = 0.0023, respectively, Fig. 3d). 24 h exposure to the stressor decreased KSRP expression with 6.25 µmol/l (p = 0.0285), 12.5 µmol/l (p = 0.003), and 25.0 µmol/l of CPA (p < 0.0001, Fig. 3d), reinforcing that KSRP is reduced in beta cell dysfunction models.

### KSRP inhibition impairs beta cell function and survival

**Figure 4:**
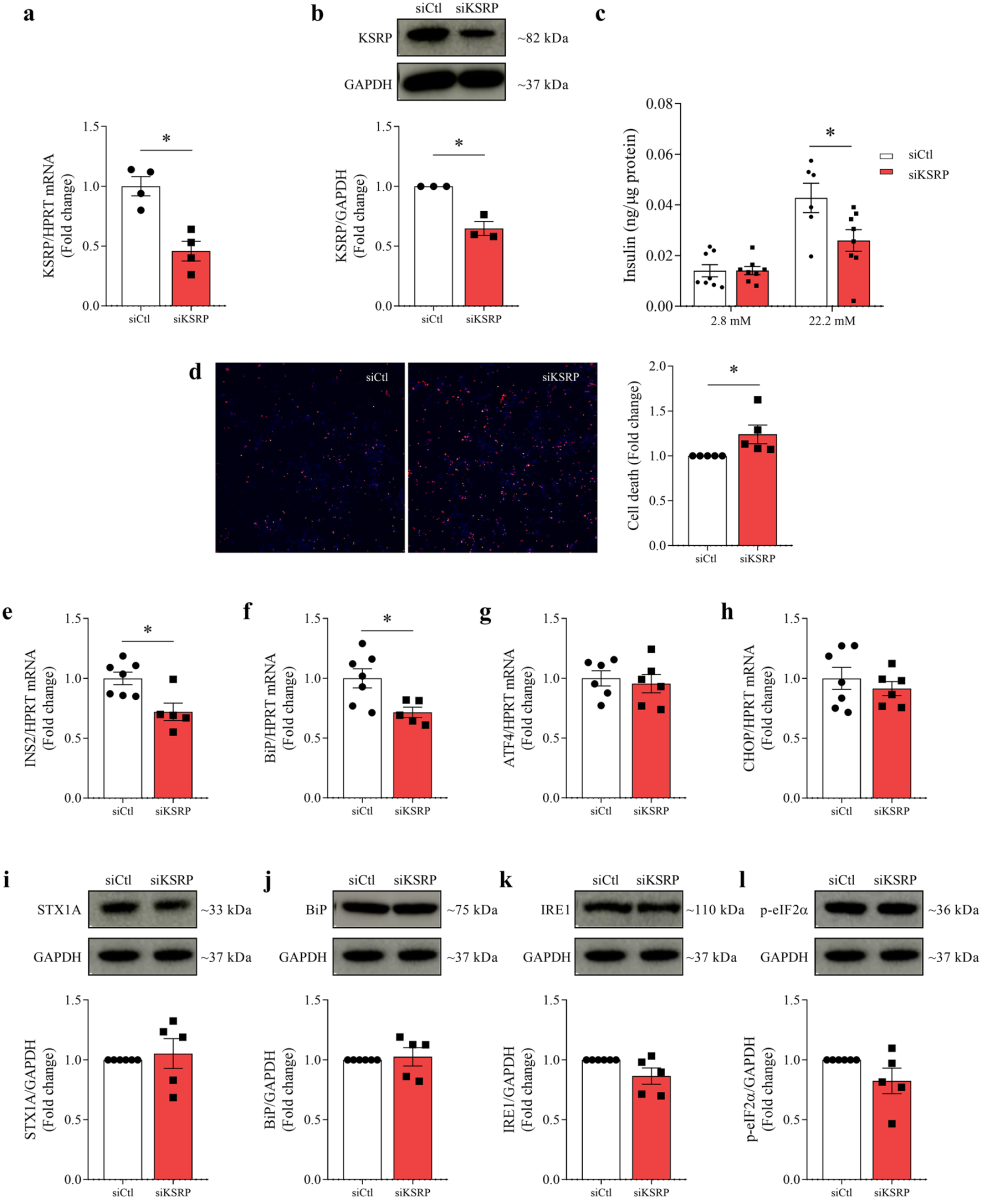
Effects of KSRP knockdown on beta cell function and survival. mRNA analysis of KSRP normalized by HPRT (**a**) and western blot analysis of KSRP normalized by GAPDH (**b**), 48 h after transfection with 70 nmol/l of siRNA using Lipofectamine RNAiMAX in INS-1E cells. Insulin secretion from INS-1E cells exposed to low (2.8 mM) and high (22.2 mM) glucose concentration (**c**). Representative images and percentage of dead cells (**d**) co-stained with Hoechst 33342 (blue) and propidium iodide (red), pictured and determined by the High Content Image System in ImageXpress Micro Confocal. mRNA expression of INS2 (**e**), BiP (**f**), ATF4 (**g**), and CHOP (**h**) normalized by HPRT in INS-1E transfected cells. Western blot analysis of STX1A (**i**), BiP (**j**), IRE (**k**), and p-eIF2α (**l**) normalized by GAPDH in INS-1E transfected cells. The results were expressed as media ± SEM and submitted to Student’s t-test. (n = 3–7). *p ≤ 0.05. Insulin secretion and cell death experiments were performed in triplicates and the other experiments in simplicate. The membranes were cut prior to exposure so that only the portion of gel containing the desired bands would be visualized. Original images for blots are presented in Supplementary Figs. S14–S16. *ATF4,* activating transcription factor 4; *BiP,* binding immunoglobulin protein; *CHOP,* C/EBP homologous protein; *GAPDH,* glyceraldehyde-3-phosphate dehydrogenase; *INS2,* insulin 2; *IRE1,* inositol-requiring enzyme 1; *HPRT,* hypoxanthine phosphoribosyltransferase; *KSRP,* KH-type splicing regulatory protein; *STX1A,* Syntaxin 1A; *p-eIF2α,* eukaryotic translation initiation factor 2α.

To investigate the involvement of KSRP on pancreatic beta cell function and survival, this protein was knocked down in INS-1E cells using short interfering RNA (siRNA). After transfection with 70 nmol/l of siRNA KSRP in INS-1E cells, a 54.2% decrease in KSRP mRNA (p = 0.0033, Fig. 4a) and a 35.2% decrease in KSRP protein content (p = 0.0038, Fig. 4b) were observed. KSRP knockdown in INS-1E cells increased beta cell death (p = 0.0499, Fig. 4d) and decreased insulin secretion in the INS-1E cells stimulated with high glucose concentration (siCtl 2.8 mM vs. siCtl 22.2 mM p = 0.0278; siKSRP 2.8 mM vs. siKSRP 22.2 mM not significant, Fig. 4c), while also causing decreased insulin gene expression (p = 0.0094, Fig. 4e). Considering ER stress is one of the most well-studied mechanisms in pancreatic beta cell dysfunction, mRNA of ER stress markers was also evaluated after the knockdown. Thus, we observed that only BiP gene expression was reduced (p = 0.0192, Fig. 4f), while other components of the pathway, such as ATF4 and CHOP, were not different between groups (Fig. 4g and h, respectively). However, the protein contents of BiP (Fig. 4j), IRE1 (Fig. 4k), and p-eIF2α (Fig. 4l) were not affected by the KSRP knockdown, nor was STX1A (Fig. 4i), which is related to the extrusion of the insulin granule.

### KSRP overexpression improves beta cell function and survival

**Figure 5:**
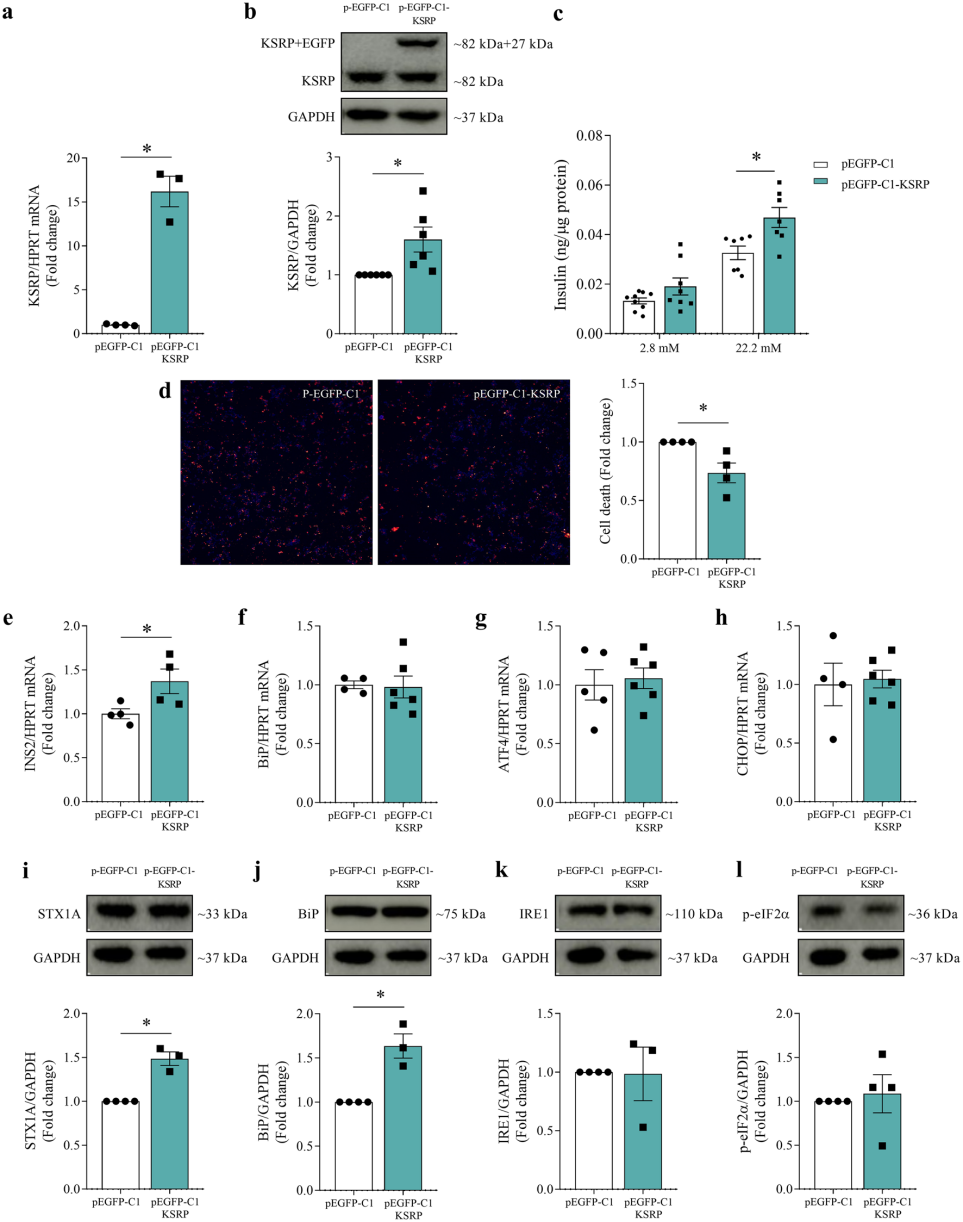
Effects of KSRP overexpression on beta cell function and survival. mRNA analysis of KSRP normalized by HPRT (**a**) and western blot analysis of KSRP normalized by GAPDH (**b**), 48 h after transfection with 500 ng of plasmid pEGFP-C1 (control) or pEGFP-C1-KSRP in INS-1E cells. Insulin secretion from INS-1E cells exposed to low (2.8 mM) and high (22.2 mM) glucose concentration (**c**). Representative images and percentage of dead cells (**d**) co-stained with Hoechst 33342 (blue) and propidium iodide (red), pictured and determined by the High Content Image System in ImageXpress Micro Confocal. mRNA analysis of INS2 (**e**), BiP (**f**), ATF4 (**g**), and CHOP (**h**) normalized by HPRT in INS-1E transfected cells. Western blot analysis of STX1A (**i**), BiP (**j**), IRE (**k**), and p-eIF2α (**l**) normalized by GAPDH in INS-1E transfected cells. The results were expressed as media ± SEM and submitted to Student’s *t* test (n = 3–6). *p ≤ 0.05. Insulin secretion and cell death experiments were performed in triplicates and the other experiments in simplicate. The membranes were cut prior to exposure so that only the portion of gel containing the desired bands would be visualized. Original blots/gels are presented in Supplementary Figs. S17, S18. *ATF4,* activating transcription factor 4; *BiP,* binding immunoglobulin protein; *CHOP,* C/EBP homologous protein; *GAPDH,* glyceraldehyde-3-phosphate dehydrogenase; *INS2,* insulin 2; *IRE1,* inositol-requiring enzyme 1; *HPRT,* hypoxanthine phosphoribosyltransferase; *KSRP,* KH-type splicing regulatory protein; *STX1A,* Syntaxin 1A; *p-eIF2α,* eukaryotic translation initiation factor 2α*.*

To promote KSRP overexpression, the INS-1E cells were transfected with a plasmid containing the KSRP sequence along with the fluorescent protein EGFP, which works as a reporter (pEGFP-C1-KSRP). Forty-eight hours after the transfection, KSRP mRNA increased by more than 1000% (p = 0.0001, Fig. 5a), and the protein expression increased by 60% (p = 0.0177, Fig. 5b). We detected the KSRP + EGFP protein band (which is approximately 32 kDa above the endogenous KSRP), confirming the effectiveness of plasmid transfection (Fig. 5b). When we evaluated KSRP overexpression in INS-1E cells, we observed opposite outcomes to KSRP inhibition. KSRP overexpression decreased beta cell death (p = 0.0207, Fig. 5d) and increased insulin secretion with high glucose concentration (pEGFP-C1 22.2 mM vs. pEGFP-C1-KSRP 22.2 mM, p = 0.0125, Fig. 5c) and insulin gene expression (p = 0.0496, Fig. 5e). Gene expression of BiP (Fig. 5f), ATF4 (Fig. 5g), and CHOP (Fig. 5h) did not show significant differences. However, KSRP overexpression increased STX1A (p = 0.0007, Fig. 5i) and BiP (p = 0.0027, Fig. 5j) protein expression, with no change in IRE1 (Fig. 5k) and p-eIF2α (Fig. 5l).

## Discussion

**Figure 6:**
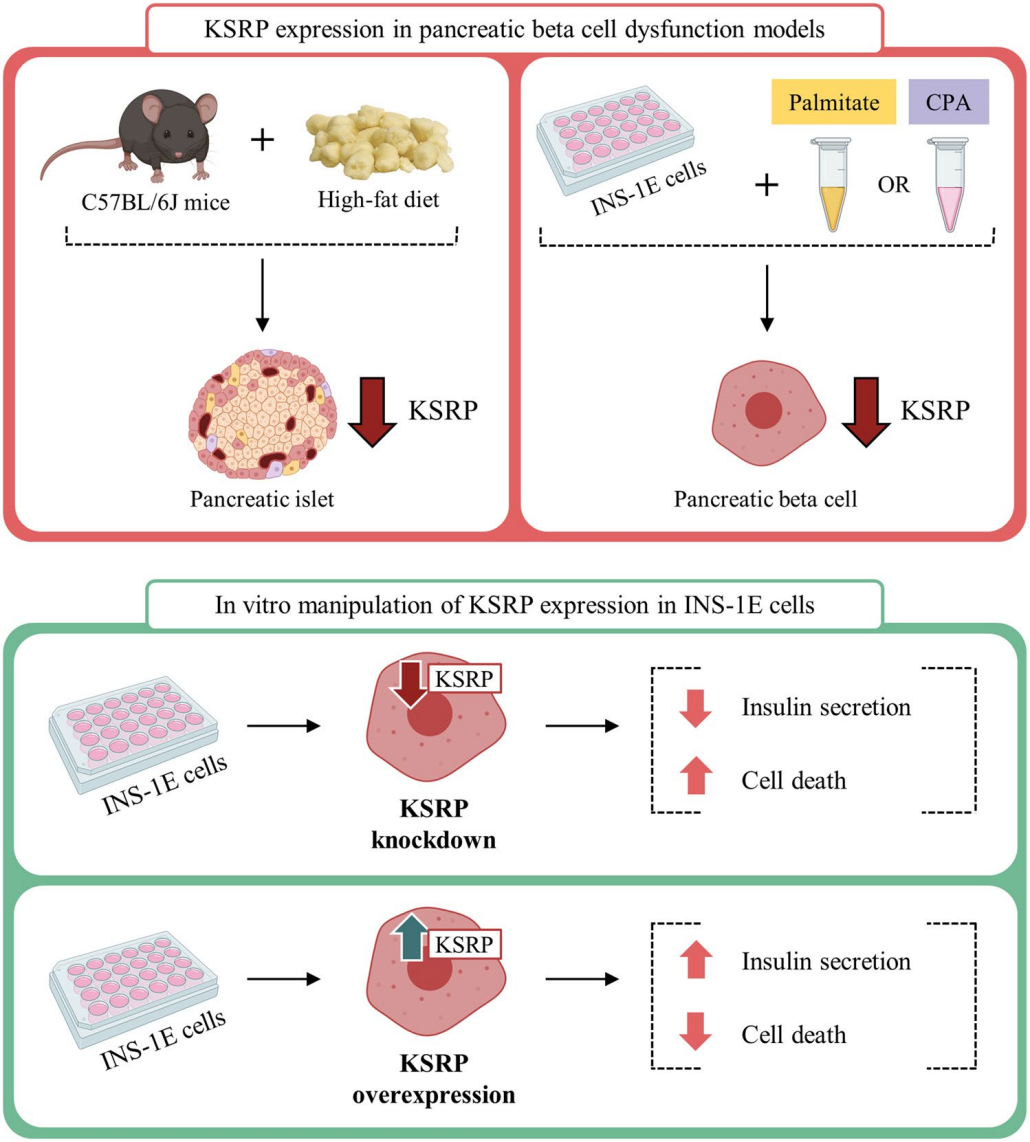
KSRP improves pancreatic beta cell function and survival. KSRP expression is reduced in obese mice pancreatic islets and in in vitro models of beta cell dysfunction, such as INS-1E exposed to CPA or palmitate. When INS-1E cells were manipulated to knock down KSRP, cell death was increased and insulin secretion was decreased. Meanwhile, when KSRP was overexpressed, cell death was decreased and insulin secretion was improved.

Here we provide evidence that KSRP plays an important role in the pancreatic beta cell. KSRP expression is reduced in beta cell overload/dysfunction models such as obese mice islets or beta cells exposed to ER stressors (palmitate and CPA) in vitro. In addition, when manipulated to knock down or overexpress KSRP, beta cells exhibit changes in insulin secretion and cell death. Based on these findings we propose KSRP is essential for beta cell normal function and survival (Fig. 6).

In pre-diabetes and in compromised metabolic states (i.e. obesity, insulin resistance, and glucose intolerance), beta cells can synthesize and secrete more insulin in an attempt to compensate for insulin resistance and maintain normoglycemia^18^. However, in the long term, an overload of beta cells can cause malfunction and cell apoptosis from different mechanisms, such as ER stress^19^. Thus, although there is still more insulin being secreted in mice fed a HFD, beta cell function is usually compromised in a large percentage, correlated to the time of diet.

As diet time would be associated with greater stress and beta cell overload, we propose that the decrease in KSRP observed in the animals' islets after 16 weeks of experimental protocol would be related to this stressful condition. In fact, studies describe that after 16 weeks, mice fed a HFD show a significant amount of cell death in the islet, unlike those fed the same diet for only 8 weeks^20^. In this context, in addition to the mRNA quantification of pancreatic islets after 16 weeks of HFD, we also performed the experiments in islets from mice after 8 weeks of HFD. Interestingly, KSRP expression is not altered after 8 weeks of HFD (Fig. 1j) while after 16 weeks of HFD, KSRP expression is decreased (Fig. 1i). These findings support the idea that KSRP reduction is associated with increased stress over time of diet.

Exposure of insulin-producing beta cells to high concentrations of fatty acids has been used to mimic pancreatic beta cell dysfunction caused by a high-fat diet, commonly associated with obesity and T2D progression. Our results show that prolonged exposure of INS-1E cells to high concentrations of palmitate leads to a decrease in insulin production (INS2 gene expression) and decreased STX1A protein expression. Proteins from the SNARE complex, such as STX1A, promote the extrusion of the insulin granule^21^. Therefore, decreased syntaxin expression is related to impaired insulin secretion, as shown in a T2D model^22^.

According to Biden et al.^15^, an increase in circulating fatty acids in obese animals may potentiate insulin secretion stimulated by glucose as a compensatory mechanism in response to insulin resistance. However, increased fatty acids in the long term lead to beta cell dysfunction due to the prolonged increase in basal secretion and the depletion of its insulin reserves^15^, which is consistent with our results.

In addition, we found that in a lipotoxic environment KSRP is also decreased in INS-1E cells. Beta cell exposure to high concentrations of free fatty acids also activates cell death pathways^23^, which was confirmed in our results by increased CHOP gene expression, a protein involved in ER stress-induced cell death in beta cells after exposure to palmitate for 24 h.

Regarding the elements of the ER stress signaling, it is known that the interaction of the immunoglobulin binding protein (BiP) keeps the transducers of the unfolded protein response (UPR) inactive. However, when BiP detaches from the transducers and the UPR is activated, there is a decrease in protein translation due to increased phosphorylation of eIF2α by the protein kinase PERK and activation of ATF4 transcription^14,24^. When all UPR mechanisms to correct ER folding defects fail, cell death pathways are activated. CHOP is a downstream target of the ATF6 and PERK/eIF2α/ATF4 pathways and promotes apoptosis^24,25^.

Our results show a decrease in the KSRP protein, proportional to the severity of damage caused by the ER stress inducer CPA in INS-1E cells. CPA also increases CHOP expression and eIF2α phosphorylation. However, 24 h after the treatment, eIF2α phosphorylation was no longer increased. This may be due to negative feedback from the prolonged increase in the CHOP protein, activating phosphatases and causing the dephosphorylation of eIF2α^26^.

Overexpressing KSRP in the pancreatic beta cell increased the expression of the chaperone protein BiP without increasing the other components of the ER stress-inducing pathway. This may represent an attempt to restore beta cell homeostasis in the ER to avoid apoptosis. A 2008 study demonstrated that the increased expression of BiP in the beta cell (INS-1E) partially reduces its susceptibility to apoptosis induced by thapsigargin (another SERCA inhibitor). Cells that overexpress BiP also have higher concentrations of proinsulin, which indicates that BiP may play an important role in the biosynthesis of this hormone^27^. This corroborates our results, which show an increase in both INS2 mRNA and BiP content in beta cells overexpressing KSRP, while KSRP knockdown decreased BiP and INS2 gene expression. It is worth mentioning that KSRP overexpression increased BiP protein expression but not mRNA content, which could be explained by regulations in protein synthesis and also by the regulation of proteasomal degradation activity^28,29^.

Our results indicate that KSRP overexpression also favors insulin production, as judged by increased glucose-stimulated insulin secretion and INS2 and STX1A expression. Likewise, we observed the opposite under KSRP knockdown, with significant decreases in INS2 gene expression and insulin secretion.

Although there is no information in the literature on the mechanism of action of KSRP in the pancreatic beta cell, studies in other cell types suggest a role for this protein in cell survival and proliferation, acting in different signaling pathways^30–33^. In agreement with these findings, our study shows that KSRP knockdown increases cell death while KSRP overexpression decreases this parameter.

The mechanism by which KSRP is linked to insulin secretion and beta cell survival needs further investigation. However, our study brings for the first time KSRP in the context of beta cell dysfunction. As KSRP is involved in post-transcriptional regulation, being part of the microRNA maturation complex^5–7^, the mechanism by which this protein acts is complex and might involve several microRNAs regulating the expression of beta cell key proteins.

In conclusion, this study illustrates that the protein KSRP performs an important role in beta cell function and survival. While more research is needed to better understand the mechanism by which KSRP acts in insulin secretion and cell death, the findings presented here could be instrumental in developing novel therapeutic strategies for diabetic individuals to maintain functional beta cells.

### Supplementary Information

Supplementary Figures.

## Data Availability

The datasets generated during and/or analyzed during the current study are available from the corresponding author on reasonable request.
