# Supplementary material for: KSRP improves pancreatic beta cell function and survival
Source: Sci Rep. 2024 Mar 13;14:6136. doi: 10.1038/s41598-024-55505-8 (PMC10937633; doi:10.1038/s41598-024-55505-8)
Supplement: Supplementary file 1 — Supplementary Figures. [file 41598_2024_55505_MOESM1_ESM.pdf]

KSRP improves pancreatic beta cell function and survival

Leticia Barssotti[1], Gabriela Moreira Soares[1], Emílio Marconato-Júnior[1], Bruna Lourençoni Alves[1], Kênia Moreno de Oliveira[1], Everardo Magalhães Carneiro[1], Antonio Carlos Boschero[1], Helena Cristina de Lima Barbosa[1*].

[1] Obesity and Comorbidities Research Center (OCRC), Department of Structural and Functional Biology, Institute of Biology, University of Campinas (UNICAMP), Campinas, SP 13083864, Brazil

*Correspondence: bsampaio@unicamp.br; Tel.: +55 19 3521 0011.

Supplementary Figure S1

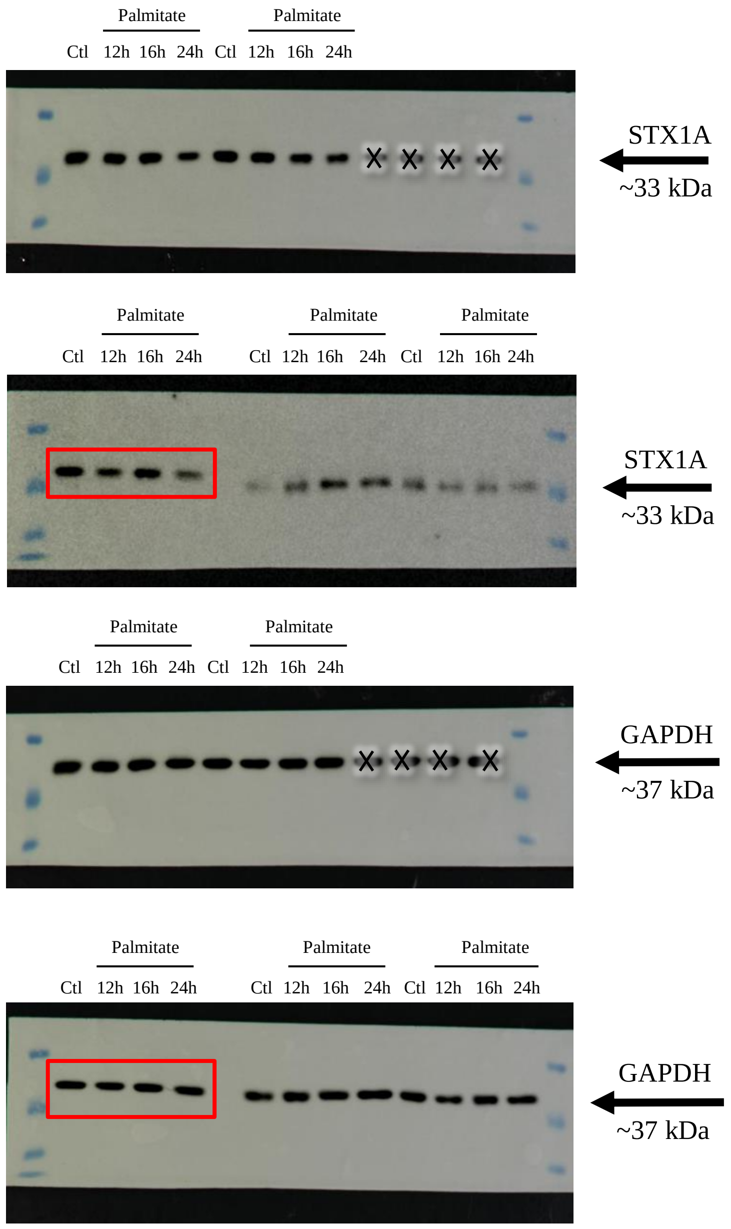

Supplementary Figure S1: Original Images for Blots from Fig. 2e. Samples are from INS-1E cells exposed to 0.5 mmol/l of palmitate (12h, 16h, or 24h). Experimental number per group = 5. The samples were transferred to nitrocellulose membranes in the sequence described in the images above. Bands marked with an X are not related to the study. The membranes were cut prior to exposure so that only the portion of gel containing the desired bands would be visualized. Red box indicate the representative image shown in Fig. 2e. GAPDH, *Glyceraldehyde-3-phosphate dehydrogenase;* STX1A, *Syntaxin 1A.*

Supplementary Figure S2

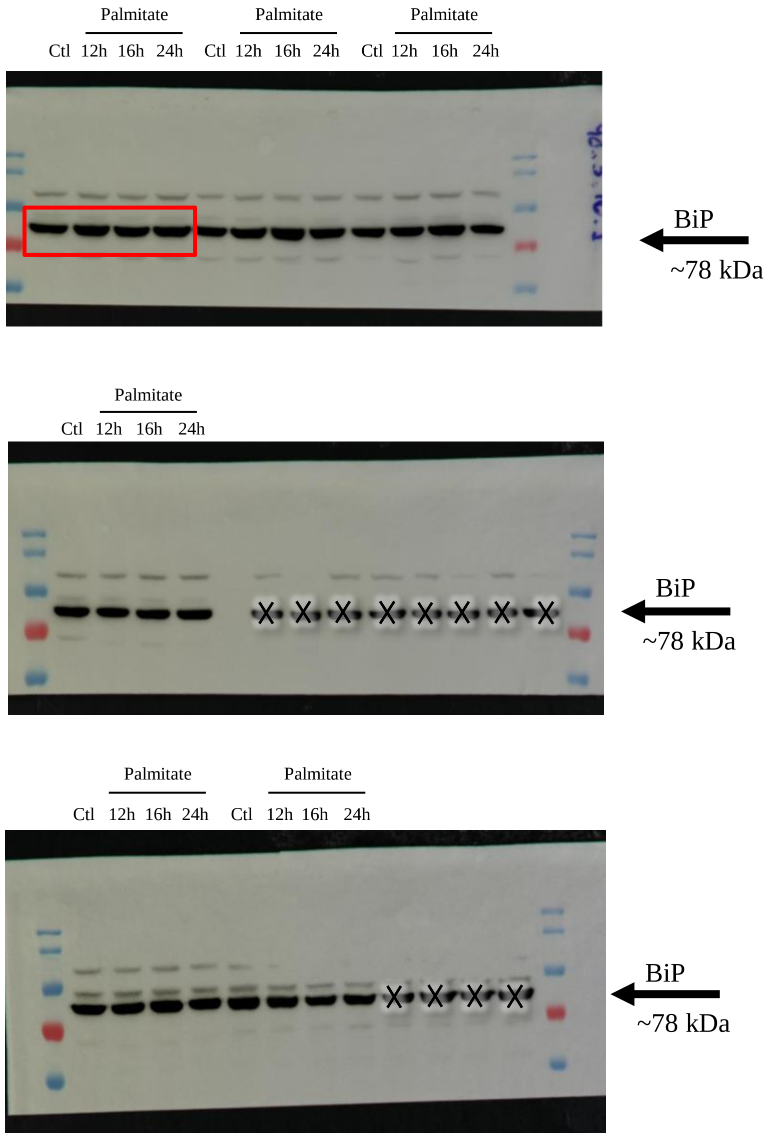

Supplementary Figure S2: Original Images for Blots from Fig. 2f. Samples are from INS-1E cells exposed to 0.5 mmol/l of palmitate (12h, 16h, or 24h). Experimental number per group = 6. The samples were transferred to nitrocellulose membranes in the sequence described in the images above. Bands marked with an X are not related to the study. The membranes were cut prior to exposure so that only the portion of gel containing the desired bands would be visualized. Red box indicate the representative image shown in Fig. 2f. BiP, *Binding immunoglobulin protein*.

Supplementary Figure S3

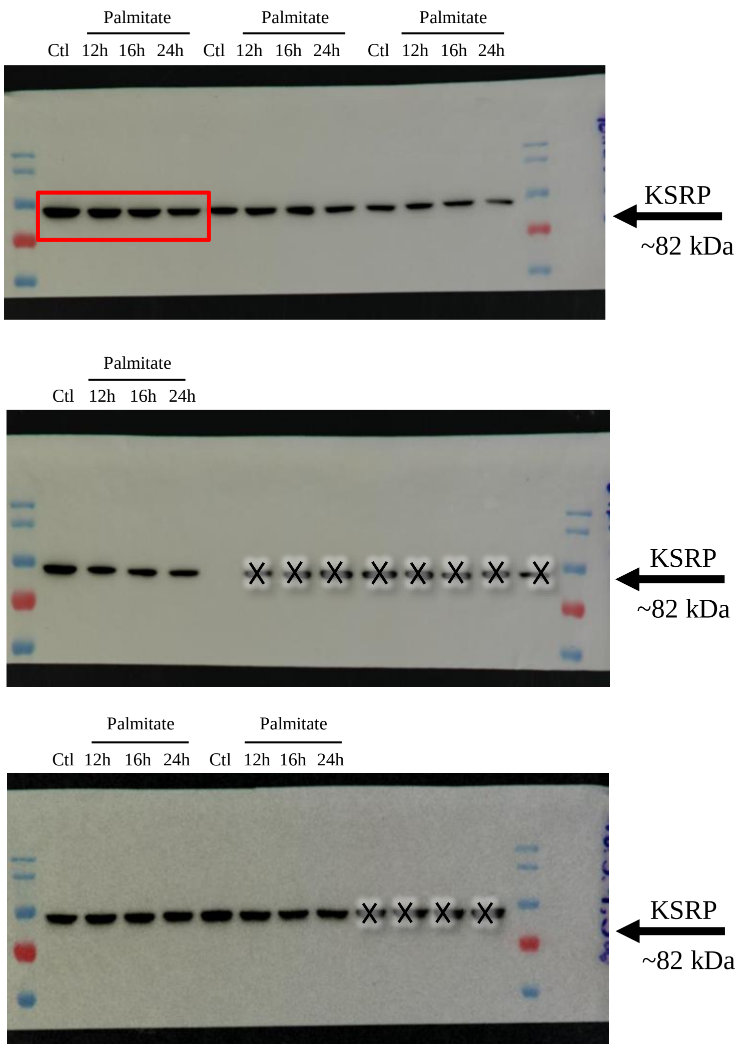

Supplementary Figure S3: Original Images for Blots from Fig. 2g. Samples are from INS-1E cells exposed to 0.5 mmol/l of palmitate (12h, 16h, or 24h). Experimental number per group = 6. The samples were transferred to nitrocellulose membranes in the sequence described in the images above. Bands marked with an X are not related to the study. The membranes were cut prior to exposure so that only the portion of gel containing the desired bands would be visualized. Red box indicate the representative image shown in Fig. 2g. KSRP, *KH-type splicing regulatory protein.*

Supplementary Figure S4

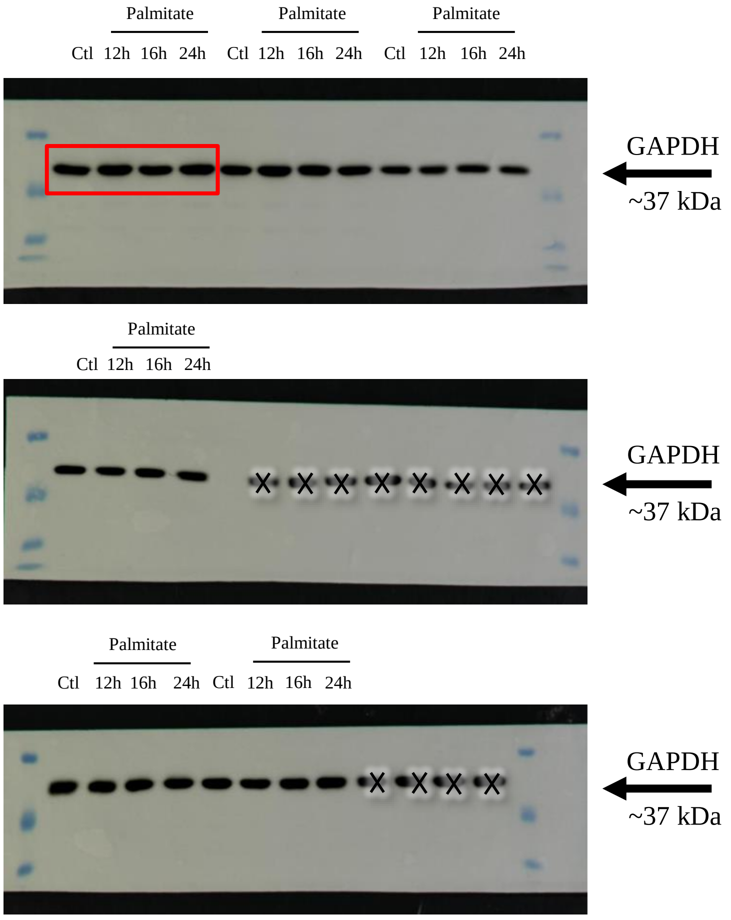

Supplementary Figure S4: Original Images for Blots from Fig. 2e, f and g. Samples are from INS-1E cells exposed to 0.5 mmol/l of palmitate (12h, 16h, or 24h). Experimental number per group = 6. The samples were transferred to nitrocellulose membranes in the sequence described in the images above. Bands marked with an X are not related to the study. The membranes were cut prior to the exposure to the antibody so that only the portion of gel containing the desired bands would be visualized. Red box indicate the representative image shown in Fig. 2e, f, and g. GAPDH, *Glyceraldehyde-3-phosphate dehydrogenase.*

Supplementary Figure S5

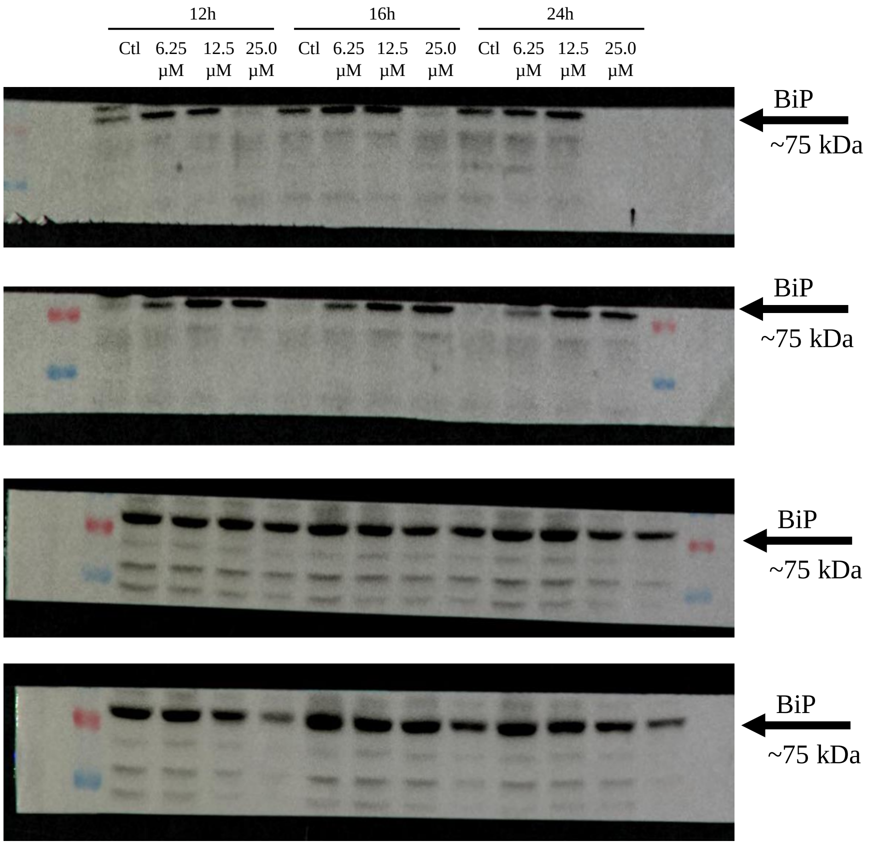

Supplementary Figure S5: Original Images for Blots from Fig. 3a. Samples are from INS-1E cells treated with 6.25 µmol/l, 12.5 µmol/l, or 25 µmol/l CPA for 12h, 16h, or 24h. The samples were transferred to nitrocellulose membranes in the sequence described in the images above. The membranes were cut prior to the exposure to the antibody so that only the portion of gel containing the desired bands would be visualized. BiP, *Binding immunoglobulin protein.*

Supplementary Figure S6

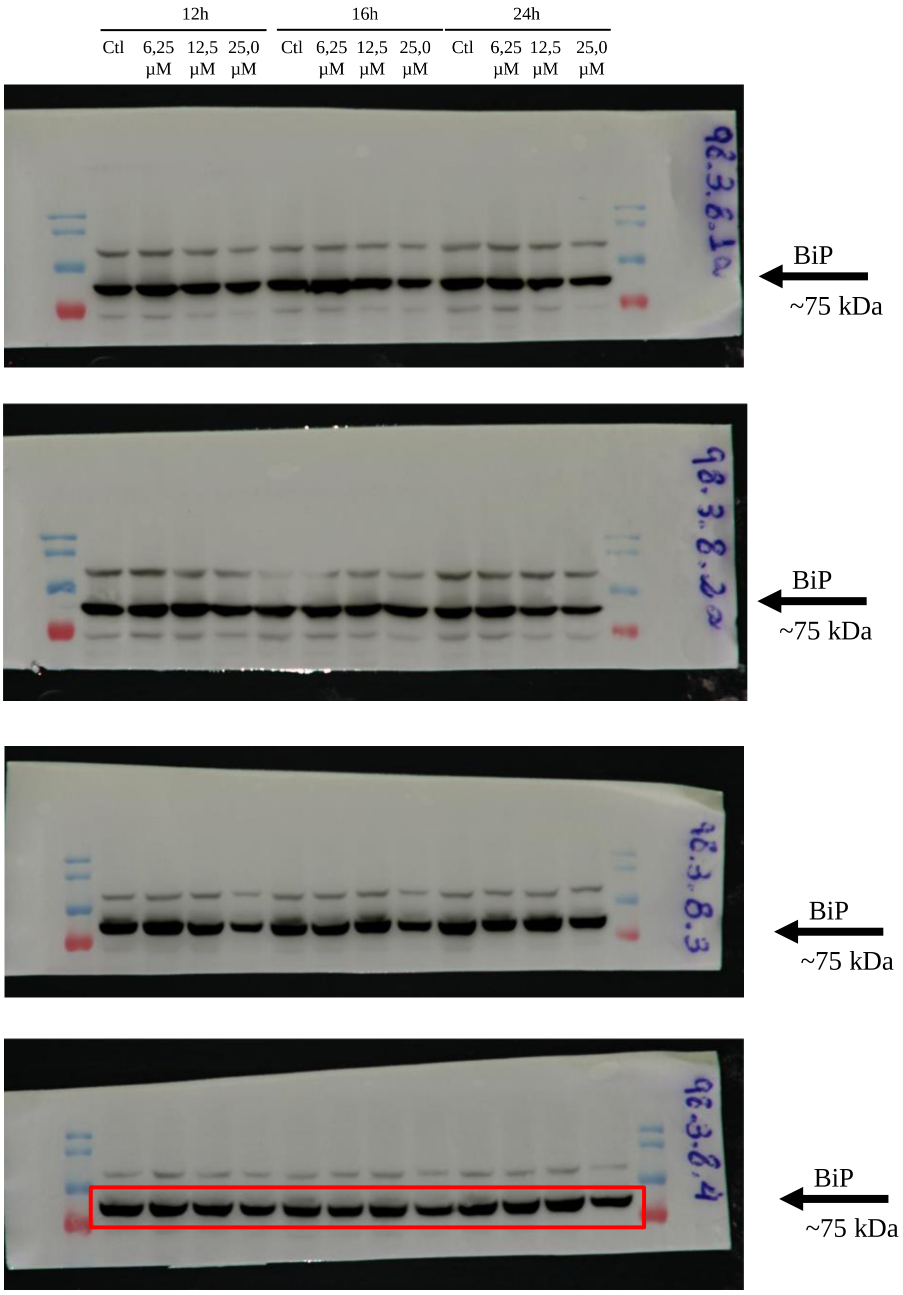

Supplementary Figure S6: Original Images for Blots from Fig. 3a. Samples are from INS-1E cells treated with 6.25 µmol/l, 12.5 µmol/l, or 25 µmol/l CPA for 12h, 16h, or 24h. The samples were transferred to nitrocellulose membranes in the sequence described in the images above. The membranes were cut prior to the exposure to the antibody so that only the portion of gel containing the desired bands would be visualized. Red box indicate the representative image shown in Fig. 3a. BiP, *Binding immunoglobulin protein.*

Supplementary Figure S7

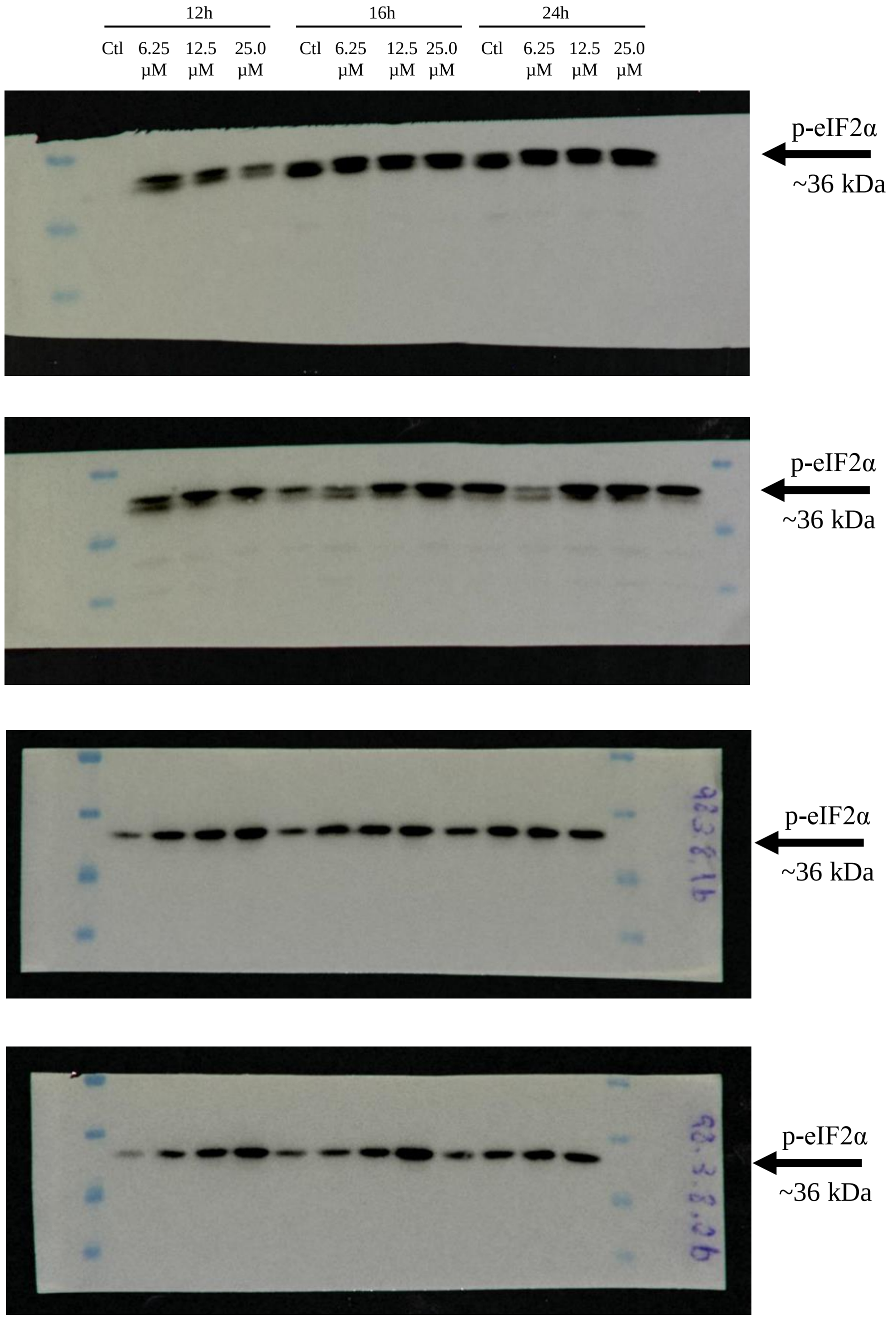

Supplementary Figure S7: Original Images for Blots from Fig. 3b. Samples are from INS-1E cells treated with 6.25 µmol/l, 12.5 µmol/l, or 25 µmol/l CPA for 12h, 16h, or 24h. The samples were transferred to nitrocellulose membranes in the sequence described in the images above. The membranes were cut prior to the exposure to the antibody so that only the portion of gel containing the desired bands would be visualized. p-eIF2α, *Eukaryotic Translation Initiation Factor 2α.*

Supplementary Figure S8

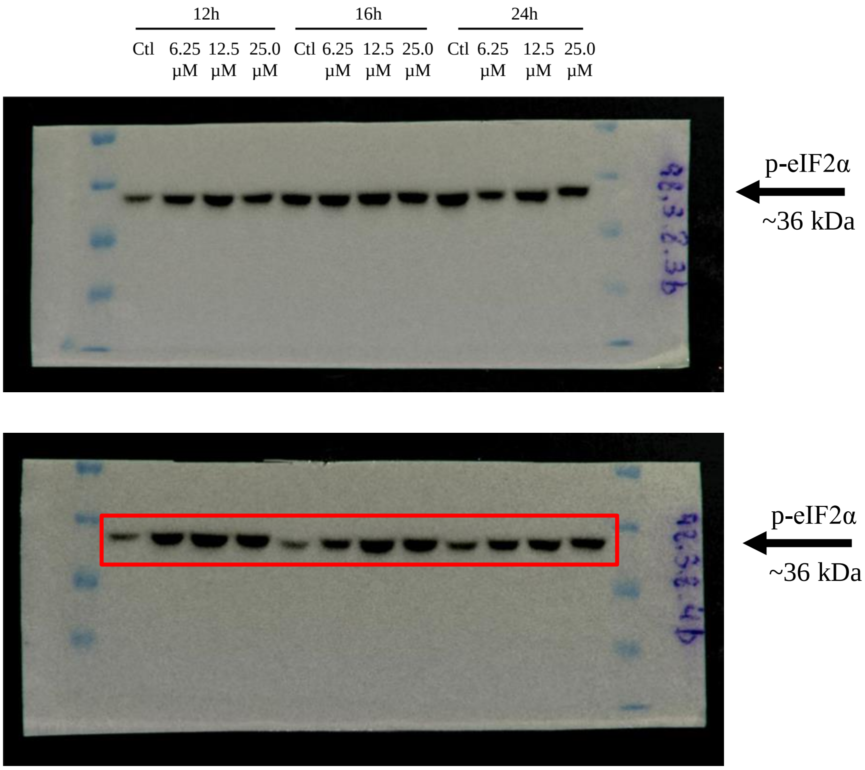

Supplementary Figure S8: Original Images for Blots from Fig. 3b. Samples are from INS-1E cells treated with 6.25 µmol/l, 12.5 µmol/l, or 25 µmol/l CPA for 12h, 16h, or 24h. The samples were transferred to nitrocellulose membranes in the sequence described in the images above. The membranes were cut prior to the exposure to the antibody so that only the portion of gel containing the desired bands would be visualized. Red box indicate the representative image shown in Fig. 3b. p-eIF2α, *Eukaryotic Translation Initiation Factor 2α.*

Supplementary Figure S9

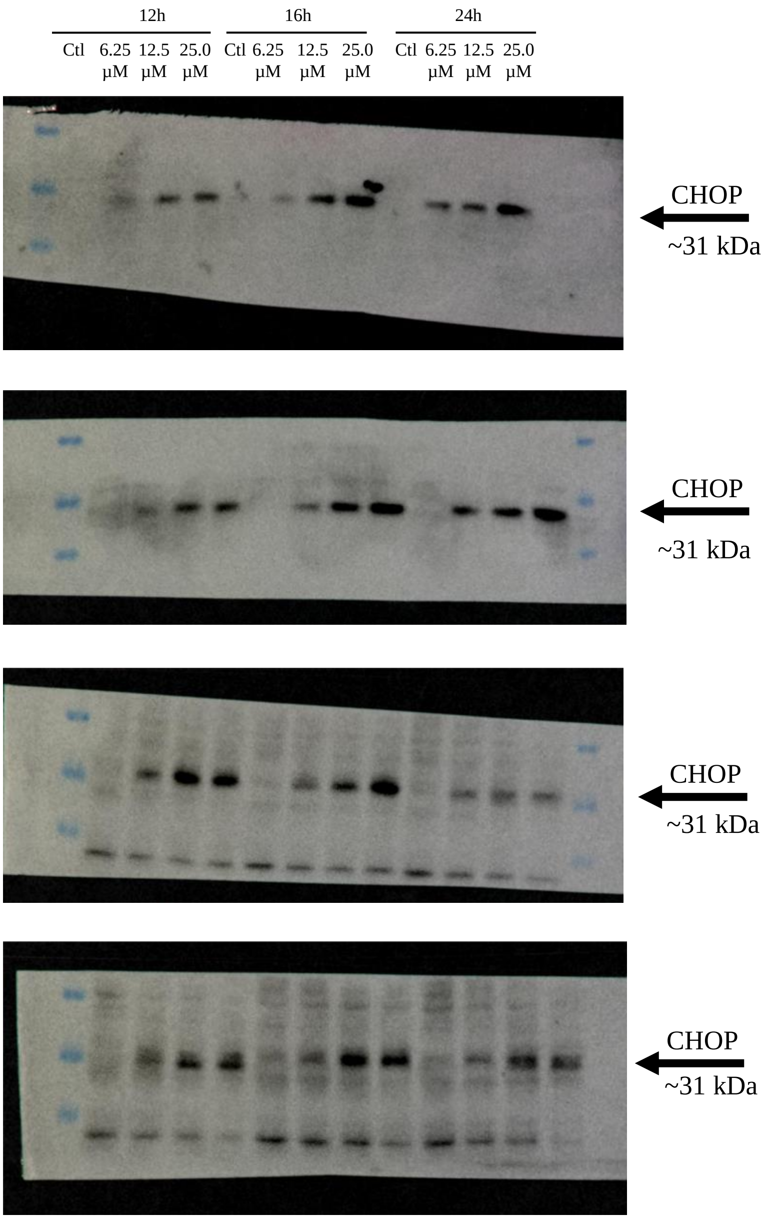

Supplementary Figure S9: Original Images for Blots from Fig. 3c. Samples are from INS-1E cells treated with 6.25 µmol/l, 12.5 µmol/l, or 25 µmol/l CPA for 12h, 16h, or 24h. The samples were transferred to nitrocellulose membranes in the sequence described in the images above. The membranes were cut prior to the exposure to the antibody so that only the portion of gel containing the desired bands would be visualized. CHOP, *C/EBP homologous protein.*

Supplementary Figure S10

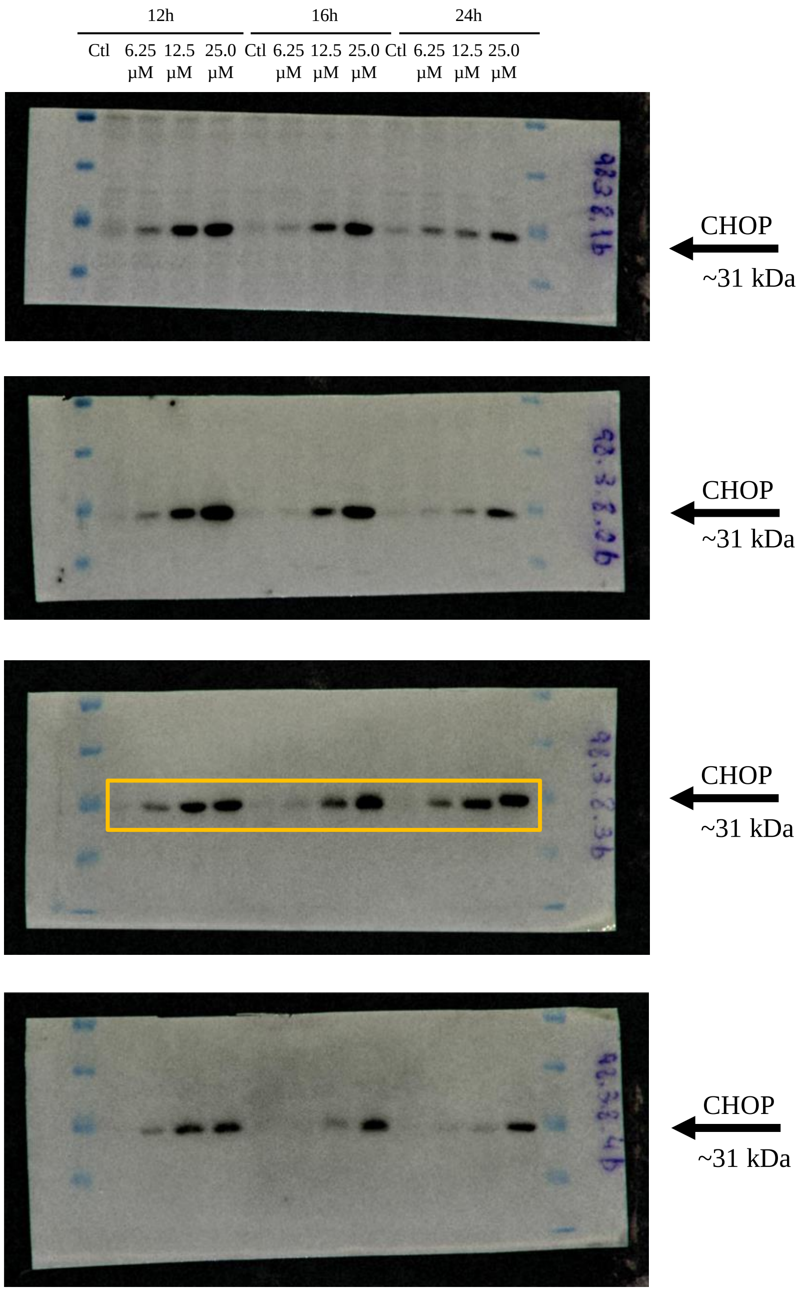

Supplementary Figure S10: Original Images for Blots from Fig. 3c. Samples are from INS-1E cells treated with 6.25 µmol/l, 12.5 µmol/l, or 25 µmol/l CPA for 12h, 16h, or 24h. The samples were transferred to nitrocellulose membranes in the sequence described in the images above. The membranes were cut prior to the exposure to the antibody so that only the portion of gel containing the desired bands would be visualized. Yellow box indicate the representative image shown in Fig. 3c. CHOP, *C/EBP homologous protein.*

Supplementary Figure S11

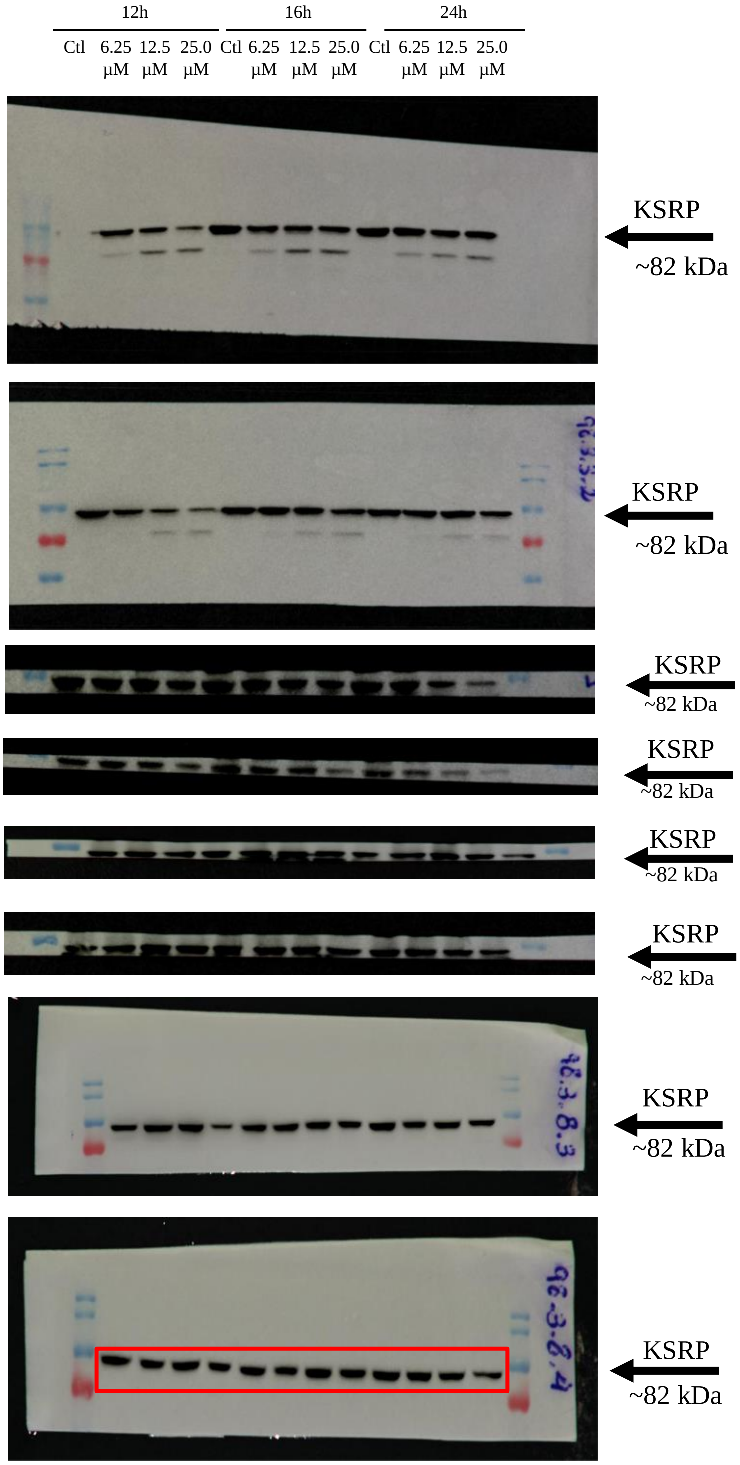

Supplementary Figure S11: Original Images for Blots from Fig. 3d. Samples are from INS-1E cells treated with 6.25 µmol/l, 12.5 µmol/l, or 25 µmol/l CPA for 12h, 16h, or 24h. The samples were transferred to nitrocellulose membranes in the sequence described in the images above. The membranes were cut prior to the exposure to the antibody so that only the portion of gel containing the desired bands would be visualized. Red box indicate the representative image shown in Fig. 3d. KSRP*, KH-type splicing regulatory protein.*

Supplementary Figure S12

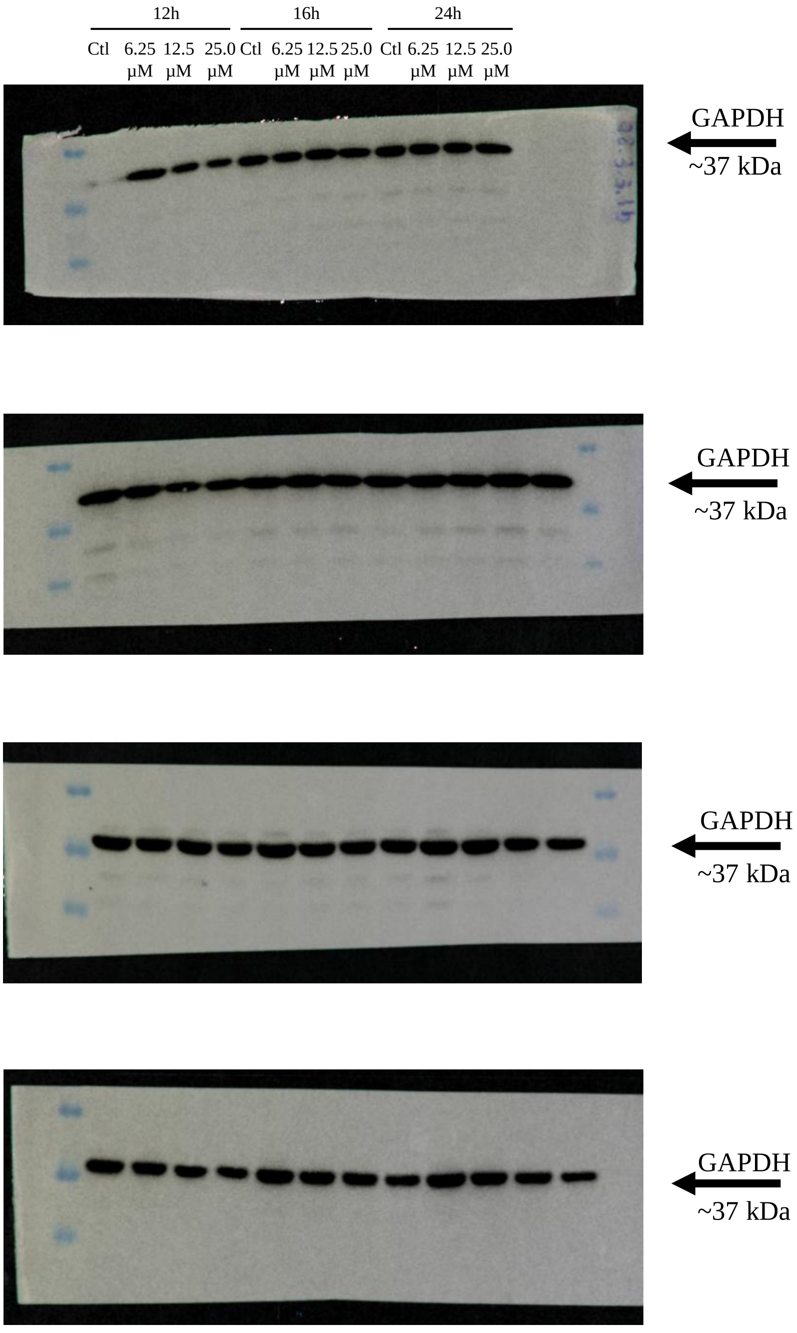

Supplementary Figure S12: Original Images for Blots from Fig. 3a, b, c and d. Samples are from INS-1E cells treated with 6.25 µmol/l, 12.5 µmol/l, or 25 µmol/l CPA for 12h, 16h, or 24h. The samples were transferred to nitrocellulose membranes in the sequence described in the images above. The membranes were cut prior to the exposure to the antibody so that only the portion of gel containing the desired bands would be visualized. GAPDH, *Glyceraldehyde-3-phosphate dehydrogenase.*

Supplementary Figure S13

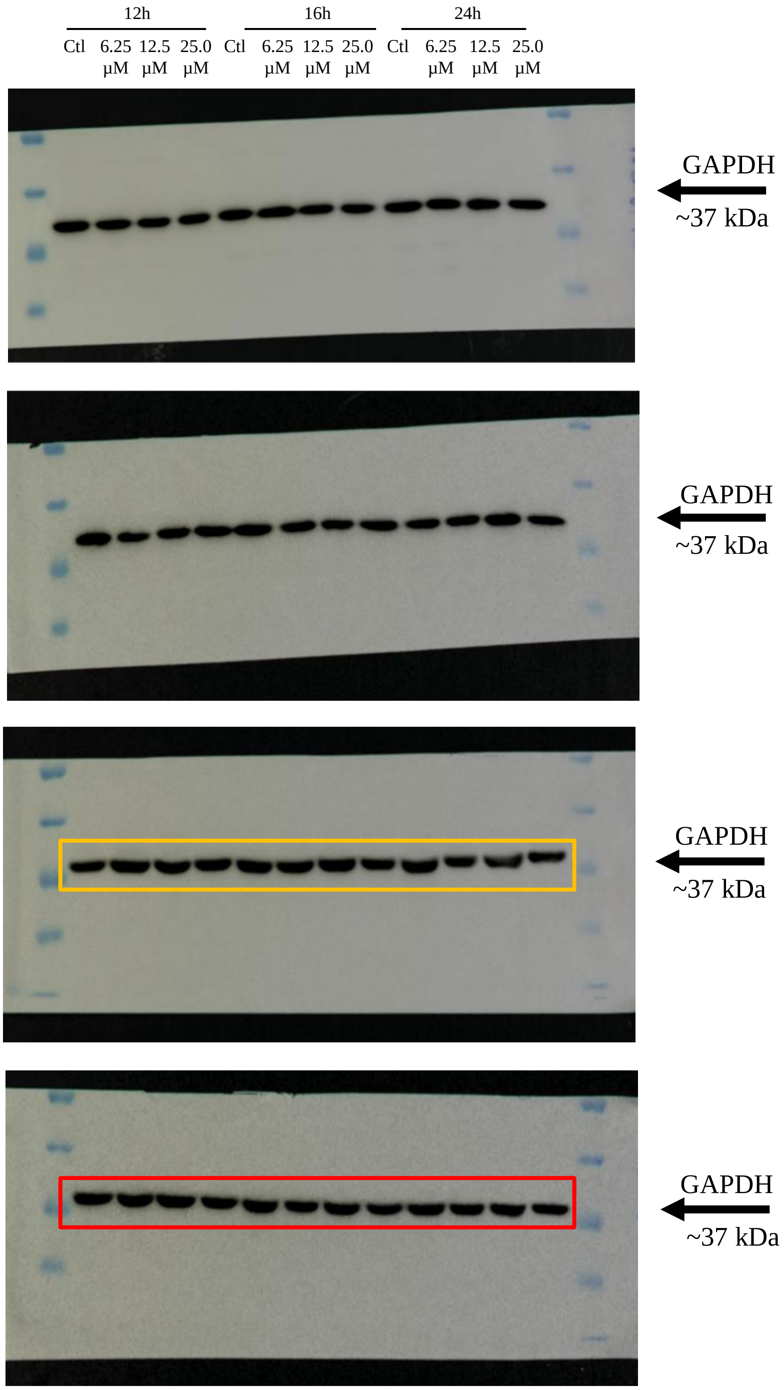

Supplementary Figure S13: Original Images for Blots from Fig. 3a, b, c, and d. Samples are from INS-1E cells treated with 6.25 µmol/l, 12.5 µmol/l, or 25 µmol/l CPA for 12h, 16h, or 24h. The samples were transferred to nitrocellulose membranes in the sequence described in the images above. The membranes were cut prior to the exposure to the antibody so that only the portion of gel containing the desired bands would be visualized. Red box indicate the representative image shown in Fig. 3a, b, and d. Yellow box indicate the representative image shown in Fig. 3c. GAPDH, *Glyceraldehyde-3-phosphate dehydrogenase.*

Supplementary Figure S14

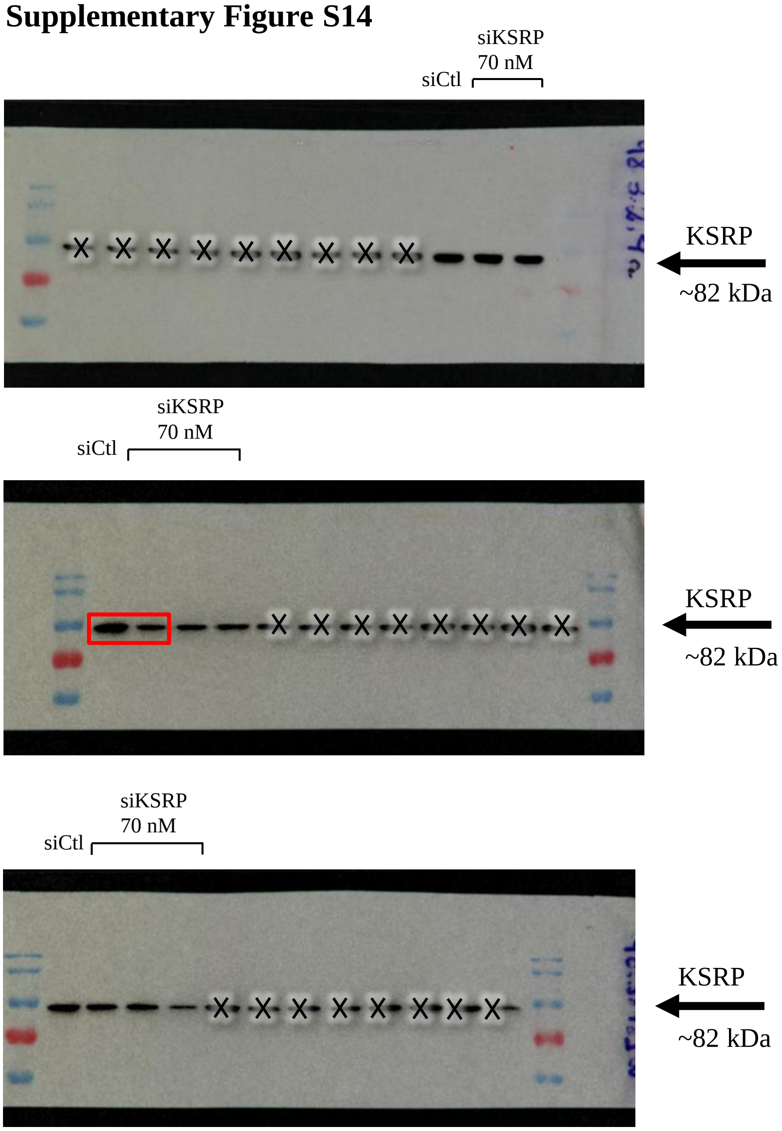

Supplementary Figure S14: Original Images for Blots from Fig. 4b. Samples are from INS-1E cells 48h after transfection with 70 nmol/l of siRNA using Lipofectamine RNAiMAX. The samples were transferred to nitrocellulose membranes in the sequence described in the images above. The membranes were cut prior to the exposure to the antibody so that only the portion of gel containing the desired bands would be visualized. Red box indicate the representative image shown in Fig. 4b. KSRP*, KH-type splicing regulatory protein.*

Supplementary Figure S15

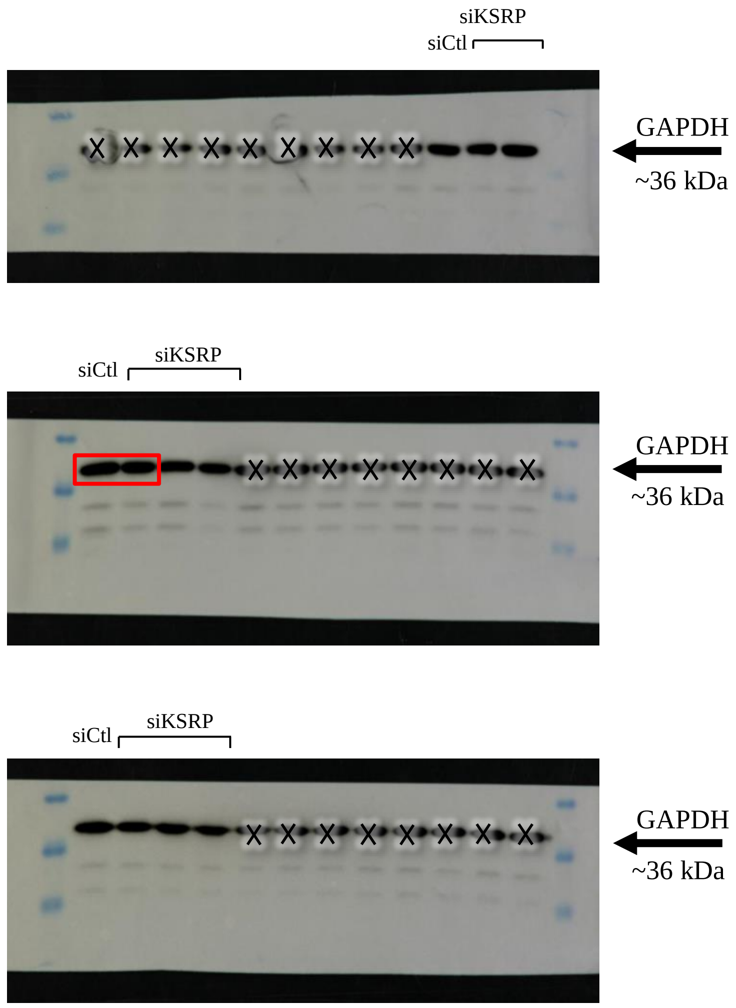

Supplementary Figure S15: Original Images for Blots from Fig. 4b. Samples are from INS-1E cells 48h after transfection with 70 nmol/l of siRNA using Lipofectamine RNAiMAX. The samples were transferred to nitrocellulose membranes in the sequence described in the images above. The membranes were cut prior to the exposure to the antibody so that only the portion of gel containing the desired bands would be visualized. Red box indicate the representative image shown in Fig. 4b. GAPDH, *Glyceraldehyde-3-phosphate dehydrogenase.*

Supplementary Figure S16

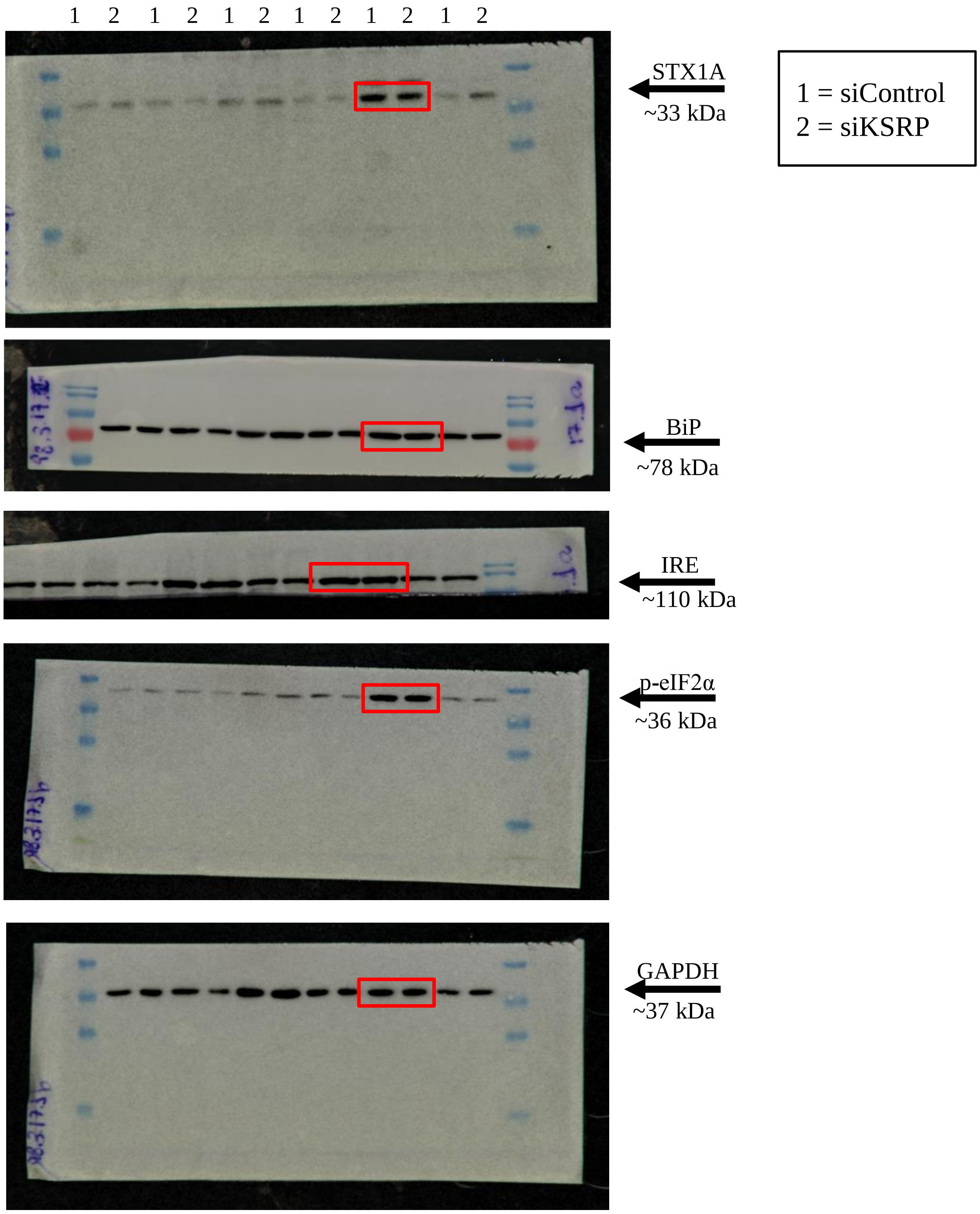

Supplementary Figure S16: Original Images for Blots from Fig. 4i, j, k, and l. Samples are from INS-1E cells 48h after transfection with 70 nmol/l of siRNA using Lipofectamine RNAiMAX. The samples were transferred to nitrocellulose membranes in the sequence described in the images above. The membranes were cut prior to the exposure to the antibody so that only the portion of gel containing the desired bands would be visualized. Red box indicate the representative image shown in Fig. 4i, j, k, and l. BiP, *Binding immunoglobulin protein*; GAPDH, *Glyceraldehyde-3-phosphate dehydrogenase*; IRE1, *Inositol-requiring enzyme 1*; STX1A, *Syntaxin 1A*; p-eIF2α, *Eukaryotic Translation Initiation Factor 2α.*

Supplementary Figure S17

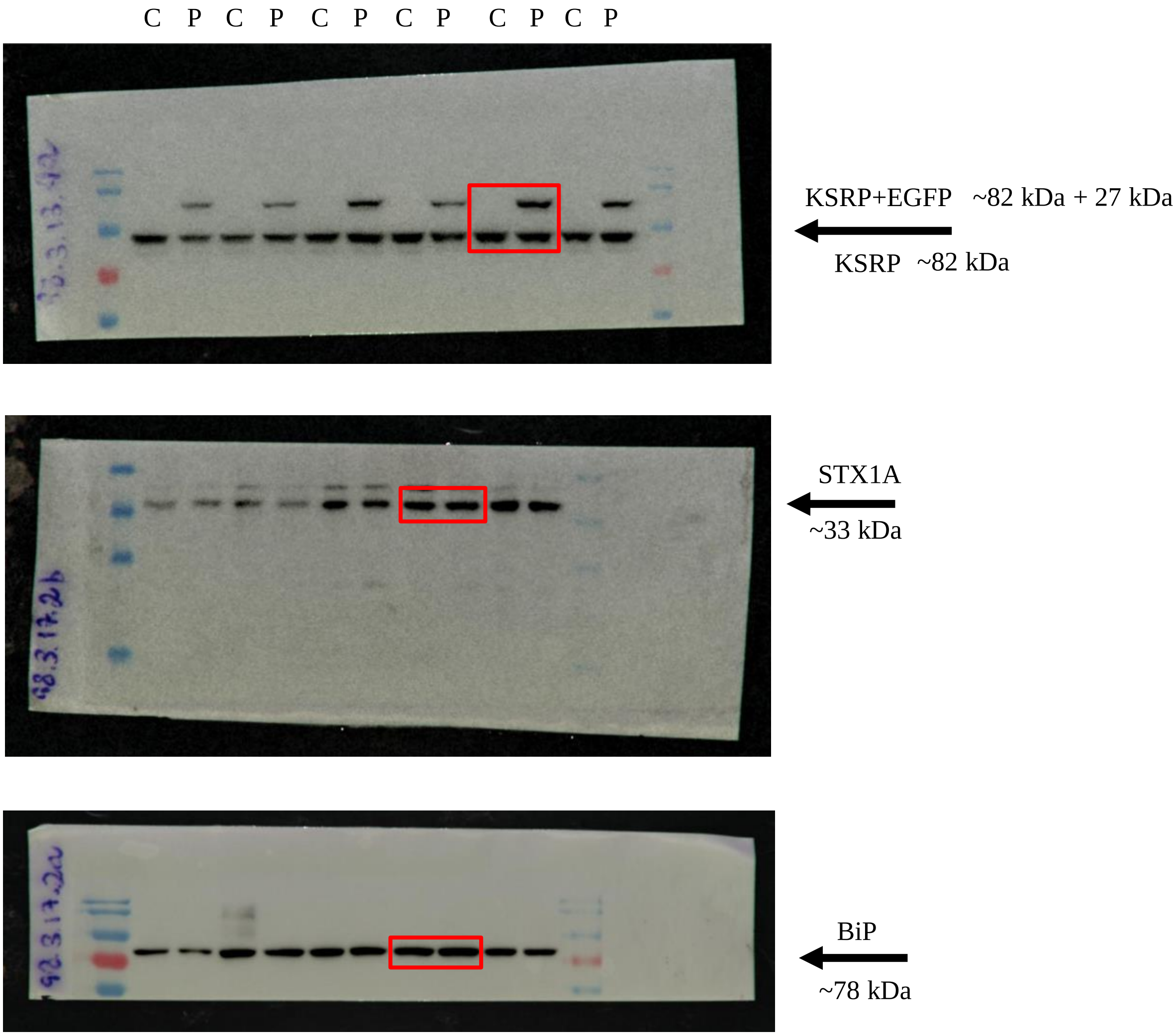

C = p-EGFP-C1 (plasmid control)
P = p-EGFP-C1 KSRP (plasmid KSRP)

Supplementary Figure S17: Original Images for Blots from Figure 5b, i, and j. Samples are from INS-1E cells 48h after transfection with 500 ng of plasmid pEGFP-C1 (control) or pEGFP-C1-KSRP. The samples were transferred to nitrocellulose membranes in the sequence described in the images above. The membranes were cut prior to the exposure to the antibody so that only the portion of gel containing the desired bands would be visualized. Red box indicate the representative image shown in Fig. 5b, i, and j. BiP, *Binding immunoglobulin protein*; KSRP, *KH-type splicing regulatory protein*; STX1A, *Syntaxin 1A*.

Supplementary Figure 18

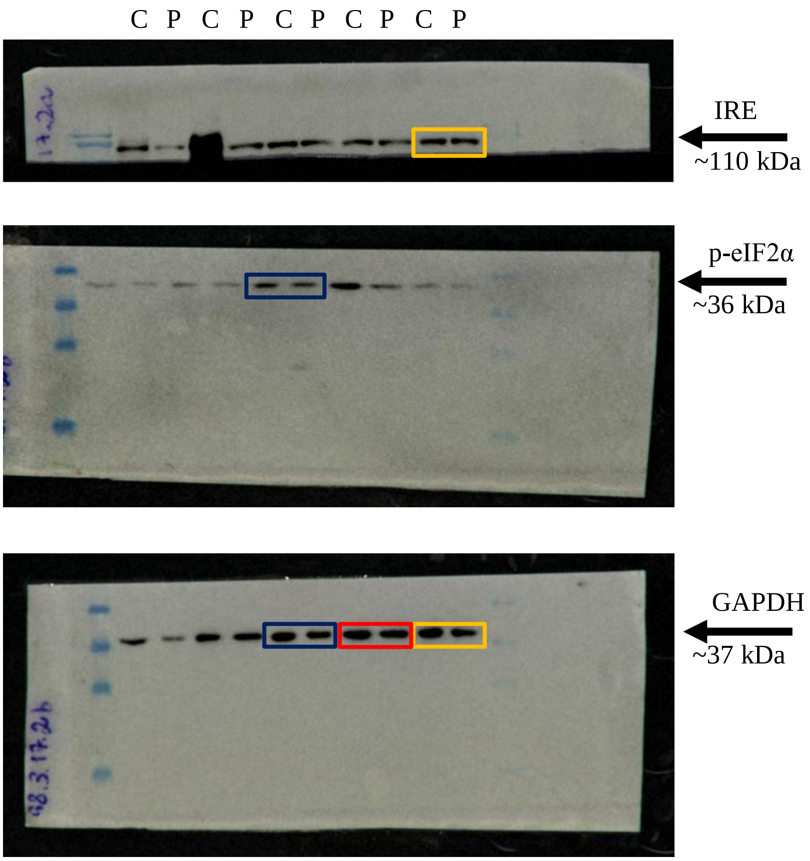

C = p-EGFP-C1 (plasmid control)
P = p-EGFP-C1 KSRP (plasmid KSRP)

Supplementary Figure S18: Original Images for Blots from Fig. 5b, i, j, k, and l. Samples are from INS-1E cells 48h after transfection with 500 ng of plasmid pEGFP-C1 (control) or pEGFP-C1-KSRP. The samples were transferred to nitrocellulose membranes in the sequence described in the images above. The membranes were cut prior to the exposure to the antibody so that only the portion of gel containing the desired bands would be visualized. Red box indicate the representative image shown in Fig. 5b, i, and j. Yellow box indicate the representative image shown in Fig. 5k. Blue box indicate the representative image shown in Fig. 5l. GAPDH, *Glyceraldehyde-3-phosphate dehydrogenase;* IRE1, *Inositol-requiring enzyme 1*; p-eIF2α, *Eukaryotic Translation Initiation Factor 2α.*
